# Cannabis Use and Misuse Following Recreational Cannabis Legalization

**DOI:** 10.1001/jamanetworkopen.2025.6551

**Published:** 2025-04-23

**Authors:** André J. McDonald, Amanda Doggett, Kyla Belisario, Jessica Gillard, Jane De Jesus, Emily Vandehei, Laura Lee, Jillian Halladay, James MacKillop

**Affiliations:** 1 Peter Boris Centre for Addictions Research, St Joseph’s Healthcare Hamilton. Hamilton, Ontario, Canada; 2 Department of Psychiatry and Behavioural Neurosciences, McMaster University, Hamilton, Ontario, Canada; 3 Michael G. DeGroote Centre for Medicinal Cannabis Research, McMaster University, Hamilton, Ontario, Canada; 4 School of Nursing, McMaster University, Hamilton, Ontario, Canada

## Abstract

**Importance:**

An increasing number of jurisdictions have legalized recreational cannabis for adults, but most evaluations have used repeated cross-sectional designs, preventing examination of within-person and subgroup trajectories across legalization.

**Objective:**

To examine changes in cannabis use and misuse in the 5 years following legalization in Canada both overall and by prelegalization cannabis use frequency using a longitudinal design.

**Design, Setting, and Participants:**

This prospective cohort study included data from community-dwelling adults who participated in up to 11 biannual assessments from September 2018 to October 2023 in Ontario, Canada. Data were analyzed from November 2023 to January 2024.

**Exposure:**

Five years of recreational cannabis legalization (baseline wave was immediately prior to legalization).

**Main Outcome and Measures:**

Primary outcomes were cannabis use frequency and cannabis misuse, assessed using Cannabis Use Disorder Identification Test – Revised (CUDIT-R) score. Prelegalization cannabis use frequency, age, and sex were examined as moderators. Secondary outcomes included changes in cannabis product preferences over time.

**Results:**

The final cohort included 1428 community-dwelling adults aged 18 to 65 years (859 [60.2%] female; mean [SD] age, 34.5 [13.9] years). Mean retention was 90% across all waves. Linear mixed-effects modeling found a significant increase in cannabis use frequency, such that the mean proportion of days using cannabis increased by 0.35% (95% CI, 0.19% to 0.51%) per year (*P* < .001) in the overall sample (1.75% over 5 years). In contrast, CUDIT-R scores (on scale of 0 to 32) decreased significantly overall (β = −0.08 [95% CI, −0.10 to −0.06] per year; −0.4 over 5 years; *P* < .001), most notably with the onset of the COVID-19 pandemic. Interaction analyses indicated that prelegalization cannabis use frequency significantly moderated changes for both outcomes (*P* < .001). Specifically, cannabis use and misuse decreased among prelegalization frequent consumers and modestly increased among occasional users and nonusers. Cannabis product preferences shifted away from dried flower, hashish, concentrates, oil, tinctures, and topicals to edibles, liquids, and vape pens.

**Conclusions and Relevance:**

In this prospective cohort study of community-dwelling adults in Canada, cannabis use frequency increased modestly in the 5 years following legalization, while cannabis misuse decreased modestly. These changes were substantially moderated by prelegalization cannabis use, with more frequent consumers of cannabis before legalization exhibiting the largest decreases in both outcomes. Although longer-term surveillance is required, these results suggest Canadian recreational cannabis legalization was associated with modest negative and positive consequences among adults.

## Key Points

**Question:**

Did cannabis use or misuse change among adults in the 5 years following recreational cannabis legalization in Canada (overall and by prelegalization cannabis use frequency)?

**Findings:**

In this cohort study including 1428 adults, cannabis use frequency increased significantly overall while misuse decreased, with small effect sizes for both. Prelegalization cannabis use significantly moderated these changes.

**Meaning:**

From a public health standpoint, this cohort study found modest changes (both negative and positive) in cannabis use behaviors in the 5 years following legalization in Canada.

## Teaser

This cohort study examines changes in cannabis use and misuse in the 5 years following legalization in Canada both overall and by prelegalization cannabis use frequency.

## Introduction

An increasing number of jurisdictions have liberalized, or are considering liberalizing, the use of recreational (nonmedical) cannabis. In October 2018, Canada became the first Group of Seven country to legalize recreational cannabis use for adults, serving as an experiment for other countries considering national policy reform. Leading up to legalization in Canada, there were concerns that cannabis use and misuse would increase due to easier access, growing social acceptability, declining perception of harm, product diversification, and increasing potency.^1,2,3,4^ Since legalization, some evidence indicates that these concerns were justified,^5,6^ although other studies have identified only limited negative outcomes.^7,8^

In general, previous research evaluating the impact of recreational cannabis legalization on cannabis use and misuse has found mixed results. For example, a repeated cross-sectional survey in the US found that states that legalized recreational cannabis use found significant postlegalization increases in the prevalence of cannabis use, frequent cannabis use, and cannabis use disorders among adults aged 26 years and older but no significant changes among young adults aged 18 to 25 years.^9^ However, another repeated cross-sectional study found that increasing cannabis use in the US was associated with general period effects and not legalization.^8^ In Canada, repeated cross-sectional studies predominantly suggest that legalization has been associated with increases in the prevalence of cannabis use and misuse among adults.^5,10,11,12^ Health care utilization studies have also suggested that cannabis-related emergency department visits and hospitalizations have increased, including cannabis use disorders, poisonings from edibles, cannabis-induced psychosis, self-harm involving cannabis, and other cannabis-attributable conditions.^13,14,15,16,17,18,19,20^

An important limitation of the cannabis legalization literature is that most previous studies have used a repeated cross-sectional design, which does not allow for the examination of within-person changes from before to after legalization. Very few within-person studies have evaluated the impact of legalization on cannabis use and misuse, representing a significant research gap.^21,22,23^ Longitudinal designs are necessary to evaluate within-person changes, as well as subgroup trajectories across legalization, such as prelegalization cannabis use levels, sex, and age. Another significant limitation of the current literature is that most studies focus on the early stages of legalization; it is widely acknowledged that understanding the impacts of legalization requires a longer postlegalization time frame.^7,24,25^

This study aimed to examine changes in cannabis use and misuse over the 5 years after legalization in a nonclinical community-based cohort of adults in Canada, with repeated measures collected approximately every 6 months. The original wave of data collection took place during the month prior to legalization, and an initial report on the 12-month outcomes found small increases in use and misuse.^22^ The current study’s first objective was to examine changes in cannabis use and misuse over a full 5 years since recreational legalization in Canada. The second objective was to examine whether prelegalization cannabis use frequency moderated these changes, that is, whether trajectories differed based on how frequently one used cannabis before legalization. This permitted evaluating whether exacerbations were present among already frequent consumers. The third objective was to examine whether cannabis product type preferences among active users changed over the 5 years since legalization.

## Methods

All procedures in this cohort study were approved by the Hamilton Integrated Research Ethics Board. All participants provided written informed consent. This study is reported following the Strengthening the Reporting of Observational Studies in Epidemiology (STROBE) reporting guidelines.

### Cohort and Study Design

Participants were recruited from an existing registry of ambulatory community-dwelling adults at St Joseph’s Healthcare Hamilton in Hamilton, Ontario, Canada. Eligibility criteria included being aged between 18 and 65 years at baseline, minimum ninth-grade education, willingness to consider participation in research studies, and no extant terminal illness. Registry enrollment involved a single extended in-person assessment.

To create the cannabis legalization surveillance cohort, registry members were invited to enroll in a supplementary study comprising periodic online assessments, with the baseline occurring in the month prior to the legalization of recreational cannabis for adults on October 17, 2018 (from mid-September to mid-October 2018). Participants were required to accept the invitation and provide informed consent. Subsequent waves of data collection were conducted each April and October up to October 2023 (11 waves total). Participants received an online gift card (CAD $40 [US $28, as of March 2025]) on completion of each wave. Data quality was protected using questions with unambiguously correct responses (eg, “For this item, select option B”), with participants required to answer at least 3 correctly for a given wave. Participants with fewer than 3 total follow-up observations were excluded, producing a final sample size of 1428 participants (95% overall inclusion). The cohort had a high retention rate across waves 2 to 11 (95.0%, 93.1%, 93.4%, 93.0%, 91.3%, 88.9%, 88.1%, 86.9%, 86.3%, 86.5%; mean [SD] retention rate, 90.2% [3.3 percentage points]) (eFigure 1 in Supplement 1).

### Measures

Two primary outcomes were examined: cannabis use frequency and cannabis misuse. Cannabis use frequency was measured with the following question from the Canadian Cannabis Survey^12^: “In the past 6 months, how often did you typically use cannabis?” Respondents could answer never, less than 1 day per month, 1 day per month, 2 to 3 days per month, 1 to 2 days per week, 3 to 4 days per week, 5 to 6 days per week, or daily. These categories were transformed into continuous values representing the proportion of days using cannabis; for categories with a range, we used the midpoint (ie, <1 day per month was calculated as 0.5 / 30.435 = 0.016; 1 day per month, 1 / 30.435 = 0.033; 2-3 days per month, 2.5 / 30.435 = 0.082; 1-2 days per week, 1.5 / 7 = 0.214; 3-4 days per week = 3.5 / 7 = 0.5; 5-6 days per week, 5.5 / 7 = 0.786; daily = 1). These values were then multiplied by 100 so that β coefficients would represent the percentage of days using cannabis for ease of interpretation.

Cannabis misuse was measured with the 8-item Cannabis Use Disorder Identification Test – Revised (CUDIT-R) scale, which asked about cannabis use and related behaviors over the past 6 months.^26^ The 8 items included: “How often do you use cannabis?”; “How many hours were you ‘stoned’ on a typical day when you were using cannabis?”; “How often during the last 6 months did you find that you were not able to stop using cannabis once you had started?”; “How often during the last 6 months did you fail to do what was normally expected from you because of using cannabis?”; “How often in the past 6 months have you devoted a great deal of your time to getting, using or recovering from cannabis?”; “How often during the last 6 months have you had a problem with your memory or concentration after using cannabis?”; “How often do you use cannabis in situations that could be physically hazardous, such as driving, operating machinery, or caring for children?”; and “Have you ever thought about cutting down, or stopping, your use of cannabis?” Responses were summed to generate a total CUDIT-R score, providing a continuous measure of cannabis misuse ranging from 0 to 32, with higher score indicating misuse. Both outcomes were treated as continuous variables.

The secondary set of 11 outcomes included reporting use of various cannabis products, including dried flower or leaf (smoked or vaporized), hashish, cannabis oil from a Health Canada licensed producer, liquid concentrate (eg, hash oil, butane honey oil), cannabis oil cartridges or disposable vape pens, solid concentrate (eg, shatter, budder), edibles (eg, prepared food products), liquid (eg, cola or tea), tinctures (eg, concentrated amounts ingested orally or taken under the tongue), topical ointments (eg, lotions, salves, balms applied directly to the skin), and fresh flower or leaf (eg, for juicing). Each cannabis product outcome was treated as binary (yes or no). This list of cannabis products was based on measures from the Canadian Cannabis Survey.^12^

Covariates included baseline age (treated as continuous), sex assigned at birth (male or female), race (Asian, Black/African, Indigenous, Latinx/Hispanic, Middle Eastern/West Asian, multiracial, or other race compared with White), marital status (unmarried or married), household income (<CAD $45 000, CAD $45 000 to CAD $90 000, ≥CAD $90 000[approximately <US $31 509, US $31 509 to US $63 018; and ≥US $63 108]), and education (≤high school, some postsecondary, Bachelor’s degree, postgraduate or professional degree). Race was included as a covariate because models adjusted for sociodemographics. Prelegalization cannabis use frequency (never, <monthly, monthly, weekly, or ≥daily) was examined as a moderator.

### Statistical Analysis

In the primary analyses, linear mixed-effects models (LMMs) were conducted to examine trends over time in cannabis use frequency (proportion of days using cannabis) and cannabis misuse (CUDIT-R score), controlling for baseline age, sex, race, marital status, household income, education, and prelegalization cannabis use frequency. Both outcomes were treated as continuous. The use of LMMs accounted for the cross-linked longitudinal nature of the data, capturing individual-level variation. While outcome data were not continuous by design, LMMs are robust to departures from distributional assumptions of normality,^27,28^ and studies have highlighted that ordinal or scale-type data can be appropriately treated as continuous.^28,29,30^ Complete case analysis was used for the primary analysis models, such that observations with missing outcome data were omitted. There were no missing data for any of the covariates, as only baseline values were included in the LMMs (baseline wave did not have item nonresponse for any of the covariates).

Prelegalization (baseline) cannabis use frequency was examined as a moderator for the association between time (continuous and rescaled to yearly changes) and the 2 outcomes. Age, sex, and the COVID-19 pandemic were also explored in secondary analyses. When testing age as a moderator, age was dichotomized to separate young adults (age <30 years), who typically age out of cannabis use,^31^ from middle-aged and older adults (age ≥30 years). The COVID-19 pandemic was partitioned into pre–COVID-19 (3 time points September 2018 to October 2019), the peak of the COVID-19 pandemic (4 time points April 2020 to October 2021) and finally the later stages of the pandemic and postpandemic (April 2022 to October 2023). For each outcome, only the identified covariates and the main effect of time were initially included, then a 2-way interaction between time and the moderator was added in a separate model. *F*-tests using restricted maximum likelihood estimation were used to test interactions.^32^ Interaction plots were created for all statistically significant interactions to facilitate interpretation.

An attrition analysis was performed that found that completing fewer than 2 follow-ups (those excluded) was significantly associated with baseline cannabis use frequency and income but no other study variables (eTable 1 in Supplement 1). A sensitivity analysis was also performed by modeling the outcomes using a joint analysis and bayesian imputation framework through the R package JointAI.^33^ JointAI models were not used for the primary analyses because omnibus tests could not be extracted from this model type, but coefficient estimates were consistent with the complete case analysis models (eTable 2 in Supplement 1).

In the secondary analyses, changes in cannabis product preferences among active cannabis users were examined at each wave (ie, only participants’ observations reporting cannabis use) by estimating adjusted prevalence differences using multivariable modified least-squares regression models.^34^ Time (continuous) was the independent variable and reporting use of a given cannabis product (yes or no) was the binary outcome. R software version 4.1.0 (R Project for Statistical Computing) was used to conduct all statistical analyses and create data visualizations. Two-sided *P* < .05 and 95% CIs that did not include the null were considered statistically significant. Data were analyzed from November 2023 to January 2024.

## Results

### Sample Description

Table 1 shows the baseline characteristics of the final study cohort of 1428 community-dwelling adults aged 18 to 65 years (859 [60.2%] female; mean [SD] age, 34.5 [13.9] years). The cohort included 142 Asian participants (9.9%), 20 Black/African participants (1.4%), 59 Latinx/Hispanic participants (4.1%), 28 Middle Eastern/West Asian participants (2.0%), 32 multiracial participants (2.2%), 1127 White/European participants (78.9%), and 20 participants (1.4%) identified as other race. Most participants were unmarried (975 participants [68.3%]) and highly educated (1314 participants [92.0%] with at least some postsecondary education), and 437 participants had an income of CAD $45 000 to CAD $90 000. Approximately half the sample reported no cannabis use, and half reported some use, with 229 participants (16.0%) scoring 6 or higher on the CUDIT-R indicating probable cannabis misuse (Table 1).^35^

**Table 1.** Baseline Characteristics of the Final Study Cohort

Indicator	Participants, No. (%) (N = 1428)
Sex	
Male	569 (39.8)
Female	859 (60.2)
Age, mean (SD), y	34.5 (13.9)
Race	
Asian	142 (9.9)
Black/African	20 (1.4)
Latinx/Hispanic	59 (4.1)
Middle Eastern/West Asian	28 (2.0)
Multiracial	32 (2.2)
White/European	1127 (78.9)
Other (including Indigenous)	20 (1.4)
Household income, $CAD^a^	
<45 000	442 (31.0)
45 000-90 000	437 (30.6)
≥90 000	549 (38.4)
Education	
≤High school	114 (8.0)
Some post-secondary	661 (46.3)
Bachelor’s degree	495 (34.7)
Postgraduate/professional degree	158 (11.1)
Marital status	
Unmarried	975 (68.3)
Married	453 (31.7)
Cannabis use frequency	
None	749 (52.4)
<Monthly	246 (17.2)
Monthly	168 (11.8)
Weekly	141 (9.9)
≥Daily	124 (8.7)
CUDIT-R score^b^	
No cannabis use	749 (52.5)
<6	450 (31.5)
≥6	229 (16.0)

Abbreviation: CUDIT-R, Cannabis Use Disorder Identification Test – Revised.

^a^ Approximately <US $31 509, US $31 509 to US $63 018, and ≥US $63 108.

^b^ A total CUDIT-R score of 6 or greater is used as a cutoff to indicate probable cannabis misuse.^35^

### Overall Changes in Cannabis Use and Moderation by Prelegalization Cannabis Use Frequency

**Figure 1.**
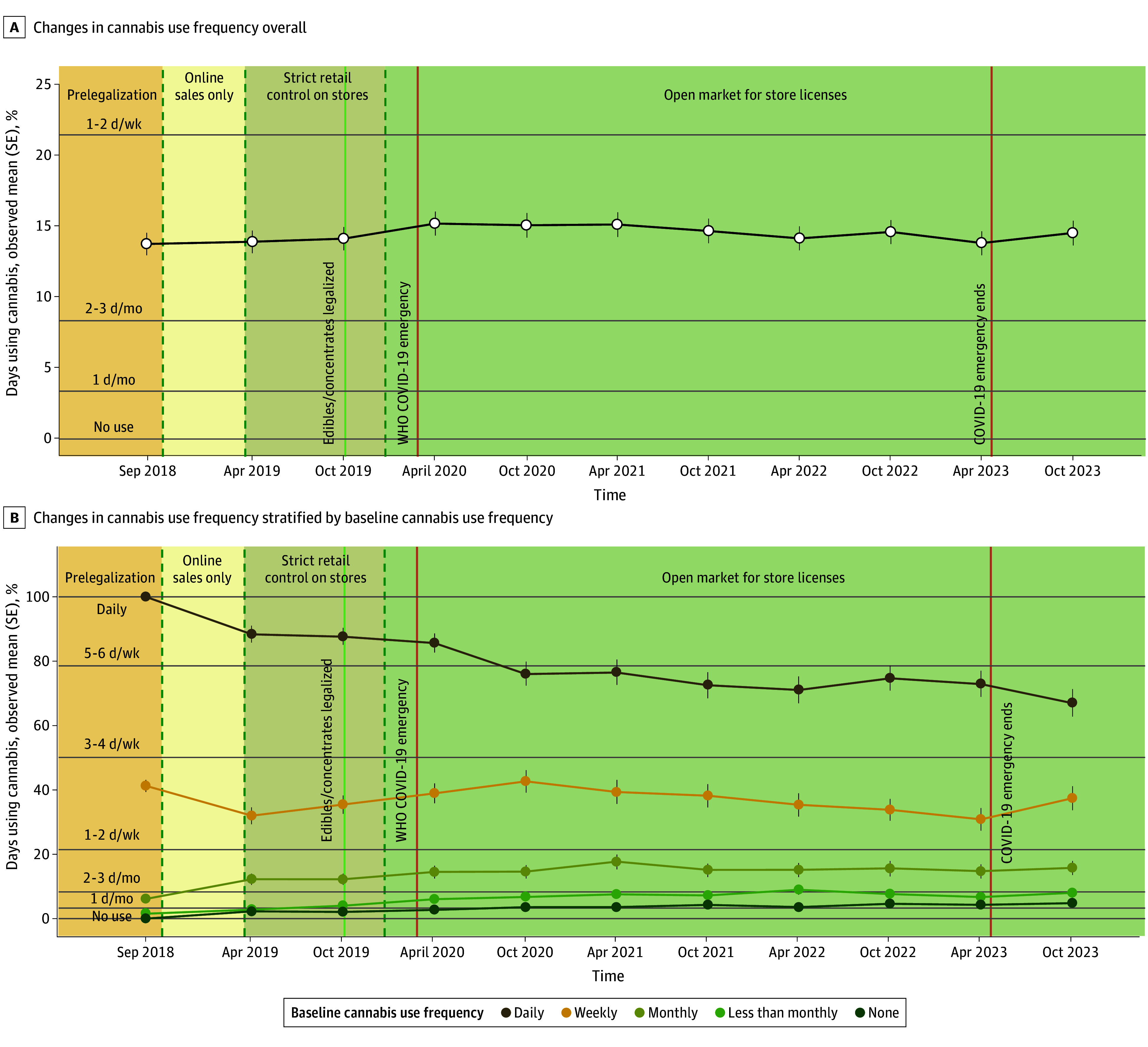
Cannabis Use Frequency Since Legalization in the Overall Sample and Stratified by Baseline Cannabis Use Frequency

Main associations of time and interactions are in Table 2. The main model found a significant increase in cannabis use frequency in the overall sample (β = 0.35; 95% CI, 0.19 to 0.51), such that the mean proportion of days using cannabis increased by 0.35% per year (ie, over 5 years, a 1.75% increase). Figure 1A shows longitudinal changes in cannabis use frequency since legalization in the overall sample.

**Table 2.** Linear Mixed Models of Time and Interaction of Prelegalization Cannabis Use Frequency for Cannabis Use Frequency and CUDIT-R^a^

Model	Cannabis use frequency^b^			CUDIT-R score^c^		
	β (95% CI)	Omnibus test		β (95% CI)	Omnibus test	
		*F*	*P* value		*F*	*P* value
Main model: time^d^	0.35 (0.19 to 0.51)	18.71	<.001	−0.08 (−0.10 to −0.06)	53.41	<.001
Interaction models						
Time^d^	0.82 (0.60 to 0.51)	19.95	<.001	0.10 (0.08 to 0.12)	379.87	<.001
Prelegalization cannabis use						
None	0 [Reference]	773.14	<.001	0 [Reference]	531.09	<.001
<Monthly	1.74 (−0.83 to 4.31)			1.50 (1.15 to 1.85)		
Monthly	9.28 (6.28 to 12.28)			3.18 (2.77 to 3.59)		
Weekly	36.74 (33.53 to 39.95)			6.18 (5.73 to 6.63)		
Daily	91.01 (87.60 to 94.42)			9.95 (9.48 to 10.42)		
Time × prelegalization cannabis use						
Time × <monthly	0.40 (−0.04 to 0.82)	121.32	<.001	−0.21 (−0.27 to −0.15)	130.71	<.001
Time × monthly	0.50 (0.01 to 0.99)			−0.32 (−0.38 to −0.26)		
Time × weekly	−1.14 (−1.69 to −0.59)			−0.50 (−0.58 to −0.42)		
Time × daily	−6.08 (−6.65 to −5.51)			−0.73 (−0.81 to −0.65)		

Abbreviation: CUDIT-R, Cannabis Use Disorder Identification Test – Revised.

^a^ All models adjusted for baseline age, sex, race, income, education, and marital status. Caution should be used in interpreting coefficients in the interaction models; eFigure 2 in Supplement 1 illustrates the interactions, facilitating interpretation.

^b^ Measured as percentage of days using cannabis.

^c^ The cannabis misuse outcome was measured using a continuous CUDIT-R score (ranging from 0 to 32).

^d^ Measured as annual change.

As shown in Table 2, a significant interaction between time and prelegalization cannabis use frequency was found (omnibus test for time × prelegalization cannabis use frequency: *P* < .001). eFigure 2 in Supplement 1 shows an interaction plot estimated from the LMM to facilitate interpretation. Figure 1B shows longitudinal changes in cannabis use frequency since legalization, stratified by prelegalization cannabis use frequency. We observed a significant decrease among individuals using daily at baseline, little change for those using weekly at baseline, and slight increases among those using monthly or less at baseline. To qualitatively characterize the patterns of change, eFigure 3 in Supplement 1 provides a person-centered alluvial plot showing transitions between cannabis use frequency groups from the first wave (before legalization in September 2018) to the last wave (5 years after legalization in October 2023). Wave-by-wave transitions are also shown in eFigure 3 in Supplement 1.

### Overall Changes in Cannabis Misuse and Moderation by Prelegalization Cannabis Use Frequency

**Figure 2.**
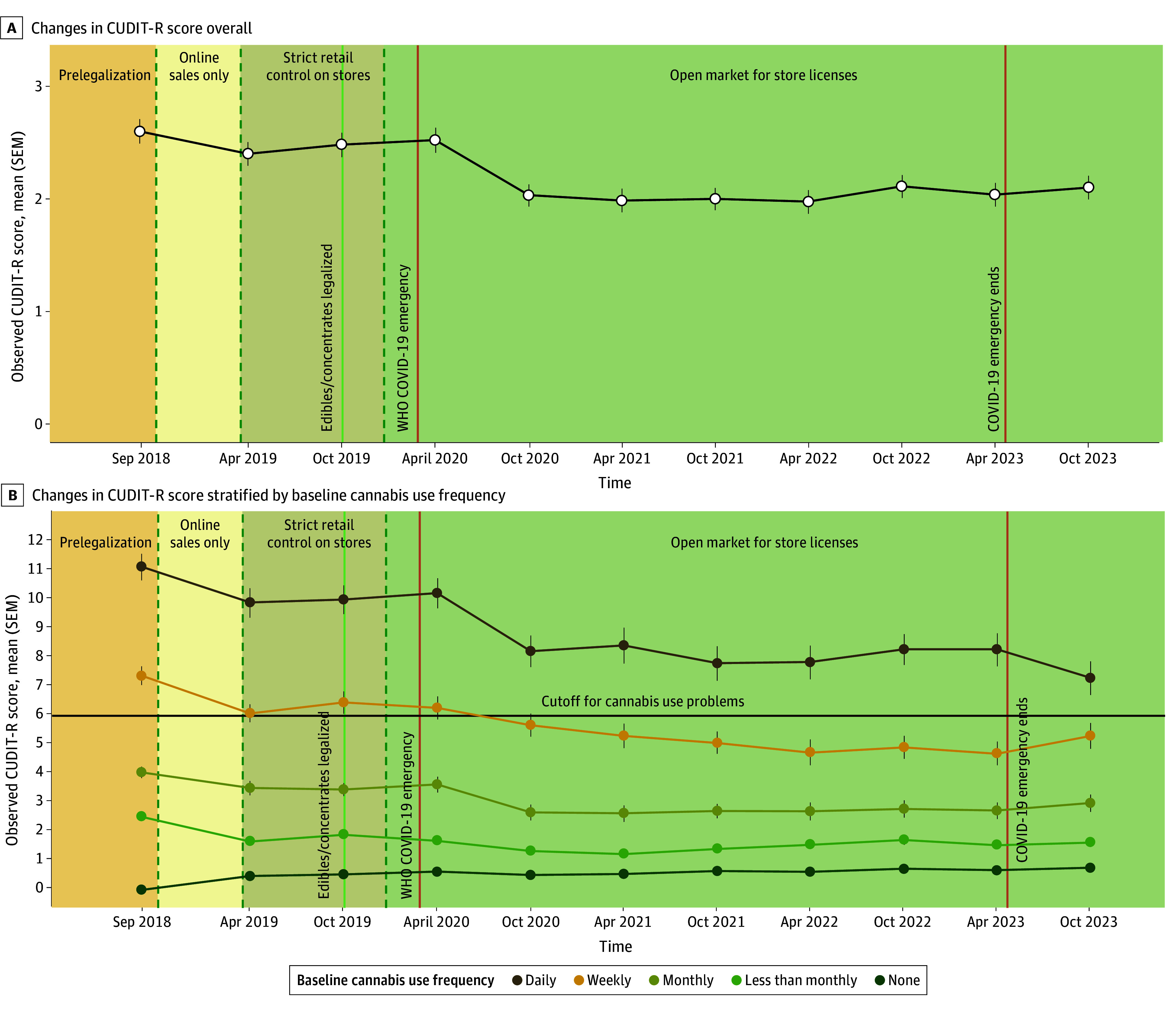
Cannabis Misuse Since Legalization in the Overall Sample and Stratified by Baseline Cannabis Use Frequency A total Cannabis Use Disorder Identification Test – Revised (CUDIT-R) score of 6 or higher is used as a cutoff to indicate probably cannabis misuse.

Results of our main model of time and interactions are presented in Table 2. The main model found a significant decrease in CUDIT-R score in the overall sample (β = −0.08; 95% CI, −0.10 to −0.06), such that the mean CUDIT-R score decreased by 0.08 points per year (ie, over 5 years, a 0.4 point decrease on average). In a follow-up analysis that removed the first item from the CUDIT-R outcome (cannabis use frequency), a similar result was found (β = −0.07; 95% CI, −0.08 to −0.05; *P* < .001). Figure 2A shows longitudinal changes in CUDIT-R score since legalization in the overall sample. Interestingly, a notable decrease was observed in cannabis misuse that occurred during the early phase of the COVID-19 pandemic (April to October 2020).

As shown in Table 2, a significant interaction between time and prelegalization cannabis use frequency was found (omnibus test for time × prelegalization cannabis use frequency: *P* < .001). eFigure 2 in Supplement 1 shows an interaction plot estimated from the LMM to facilitate interpretation. Figure 2B shows longitudinal changes in cannabis use frequency since legalization stratified by prelegalization cannabis use frequency. We observed a significant decrease among individuals using monthly or less than monthly at baseline and a slight increase among those not using at baseline. Of note, mean CUDIT-R scores among individuals using weekly at baseline crossed from above to below the validated CUDIT-R cutoff score of 6 indicating probable cannabis misuse.^35^ To qualitatively characterize the patterns of change, eFigure 4 in Supplement 1 provides a person-centered alluvial plot showing transitions between cannabis misuse groups from the first wave (before legalization in September 2018) to the last wave (5 years after legalization in October 2023). Wave-by-wave transitions are shown in eFigure 4 in Supplement 1.

### Changes in Cannabis Product Preferences

Table 3 presents results from the secondary analyses estimating adjusted prevalence differences (annual percentage changes) in cannabis product preferences among active cannabis users. Statistically significant decreases in use of dried flower, solid concentrate, liquid concentrates, cannabis oil, tinctures, topical ointments, and hashish were observed. Conversely, statistically significant increases in use of edibles, liquids, and cannabis oil cartridges or disposable vape pens were observed. The most pronounced decrease was in dried flower use, with a 3.56% (95% CI, 2.91% to 4.22%) annual decrease in prevalence among active cannabis users (from 81.3% prelegalization to 64.6% at 5 years postlegalization). The most pronounced increase was in use of cannabis oil cartridges or disposable vape pens, with a 3.39% (95% CI, 2.72 to 4.05%) annual increase in prevalence among active cannabis users (from 18.4% prelegalization to 33.0% at 5 years postlegalization). eFigure 5 in Supplement 1 presents the proportion of active cannabis users reporting use of different products over time since legalization.

**Table 3.** Adjusted Prevalence Differences in Cannabis Product Preferences Among Active Cannabis Users in the 5 Years Since Legalization Estimated From Multivariable Modified Least Squares Regression Models^a^

Cannabis product use	Annual percentage change		aPD × 5 y, %
	aPD (95% CI), %	*P* value	
Dried flower/leaf (smoked or vaporized)	−3.56 (−4.22 to −2.91)	<.001	−17.80
Solid concentrate (eg, shatter, budder)	−2.22 (−2.68 to −1.76)	<.001	−11.10
Liquid concentrate (eg, hash oil, butane honey oil)	−1.45 (−1.88 to −1.03)	<.001	−7.25
Cannabis oil from a Health Canada licensed producer	−0.95 (−1.53 to −0.38)	.001	−4.75
Tinctures (eg, concentrated amounts ingested orally or taken under the tongue)	−0.89 (−1.34 to −0.45)	<.001	−4.45
Topical ointments (eg, lotions, salves, balms applied directly to the skin)	−0.79 (−1.27 to −0.30)	.001	−3.95
Hashish	−0.72 (−1.15 to −0.29)	.001	−3.60
Fresh flower/leaf (eg, for juicing)	−0.08 (−0.24 to 0.07)	.29	−0.40
Edibles (eg, prepared food products)	1.22 (0.46 to 1.99)	.002	6.10
Liquid (eg, cola/tea)	2.21 (1.77 to 2.66)	<.001	11.05
Cannabis oil cartridges or disposable vape pens	3.39 (2.72 to 4.05)	<.001	16.95

Abbreviation: aPD, adjusted prevalence difference.

^a^ All models adjusted for baseline age, sex, race, income, education, and marital status, and used robust variance estimation.

### Moderation of Changes Over Time by Sex, Age, and the COVID-19 Pandemic

Sex and age were examined as moderators for change in cannabis use frequency and CUDIT-R score over time. No interactions were present for cannabis use frequency, but interactions were present for sex and age in relation to cannabis misuse (eTable 3 in Supplement 1), such that reductions were more pronounced among males and younger participants, consistent with higher cannabis misuse scores in males and younger participants. eFigure 6 in Supplement 1 shows longitudinal changes in cannabis misuse score by age. The COVID-19 pandemic was not a significant moderator for changes in cannabis use frequency but was for cannabis misuse.

Interaction plots were created for all statistically significant interactions. As illustrated in eFigure 2 in Supplement 1, age was a noticeable moderator for cannabis misuse, with younger participants starting with a higher level of cannabis misuse compared with older participants, with the gap shrinking over time. The COVID-19 pandemic was also a noticeable moderator for cannabis misuse (eFigure 2 in Supplement 1), such that cannabis misuse decreased modestly during the prepandemic period, decreased more sharply during the pandemic, and increased modestly during the postpandemic period.

## Discussion

This prospective cohort study found that cannabis use frequency modestly increased while cannabis misuse decreased modestly during the first 5 years of legalization in a community-based cohort of adults in Canada. From a public health standpoint, these results are mixed, as increased use might be considered harmful, while decreased misuse is a positive outcome. Given that age was a significant moderator, whereby younger adults had larger declines in problems, these mixed findings may partly be due to the aging-out developmental trajectory that typically occurs during young adulthood,^31^ which was more prominent for cannabis misuse than cannabis use frequency. Similar results have been observed in another cohort study,^21^ which found a significant decrease in cannabis-related adverse consequences but little change in cannabis use frequency. We note that for both outcomes, while changes were statistically significant, it is debatable whether these changes were clinically significant, particularly in the case of CUDIT-R, which decreased by only 0.4 points on a scale of 32 over 5 years.

Cannabis misuse notably declined immediately after the onset of the COVID-19 pandemic and never returned to prepandemic levels. This was most pronounced among prelegalization high-frequency consumers. This finding was confirmed when the pandemic was assessed as a moderator. Another longitudinal study similarly found that among pre–COVID-19 frequent cannabis users, cannabis use frequency increased early in the pandemic while cannabis use disorder symptom severity decreased slightly.^36^ Understanding the environmental or psychological factors leading to these changes warrants further investigation.

When examining prelegalization cannabis use as a moderator for the association between time and cannabis use frequency, a significant interaction was found such that cannabis use frequency decreased among those already using frequently prelegalization and increased among prelegalization abstainers. Changes in cannabis misuse were similarly moderated by prelegalization cannabis use frequency, such that cannabis misuse decreased for all groups already using cannabis prelegalization and increased for prelegalization abstainers. These findings do not support the notion that cannabis legalization would amplify or otherwise exacerbate existing patterns of use among active consumers. For both outcomes, it is also possible that regression to the mean explains part of the interaction findings. Fundamentally, however, these results do not suggest increased adverse outcomes for adults who were actively using cannabis before legalization.

Secondary analyses revealed significant changes in cannabis product preferences among those using cannabis actively over time, with decreases in dried flower, concentrates, cannabis oil, tinctures, topical ointments, and hashish use and increases in use of edibles, liquids, and cannabis oil cartridges or disposable vape pens. These findings are similar to those of the International Cannabis Policy Study,^5^ which also found a pronounced decrease in use of dried flower, and increases in use of edibles, oils, and drinks. Edibles and liquids only became legal in Canada on October 17, 2019, 1 year after cannabis flower and oils became legal, although availability in Canada’s largest provinces occurred in January 2020.^37,38^ That cannabis users transitioned away from dried flower, which is typically combusted, and toward noncombusted oils, edibles, and drinks may be viewed as a positive development from a lung health perspective. However, it is concerning that use of cannabis oil cartridges and disposable vape pens increased, as these products can have extremely high Δ9-tetrahydrocannabinol concentrations, which are becoming increasingly popular among youth.^39,40^ Moreover, it is potentially concerning that cannabis drinkables are becoming more popular, given how little research has examined their health effects.

This study addresses important research gaps and adds a new dimension to the small but growing cannabis legalization evaluation literature, complementing health care utilization studies and repeated cross-sectional surveys, which have mostly found negative outcomes associated with legalization for adults.^10,11,12,13,14,15,16,17,18,19,20^ To our knowledge, this study uses the longest follow-up of any within-individual longitudinal study evaluating recreational cannabis legalization, providing a more nuanced understanding of cannabis behavior changes following legalization. The cohort had a high retention rate, with a mean of 90% retention across all follow-up waves, with 87% remaining at the 5-year mark.

### Limitations

This study has some limitations. Without comparison to a jurisdiction without cannabis legalization, it is difficult to attribute changes in cannabis use and misuse to legalization per se. It is possible that age-related decreases partly explained the interactions observed. There was only 1 prelegalization time point, which precluded a more comprehensive age-period-cohort analysis. It is also difficult to disentangle the impact of the COVID-19 pandemic on cannabis use behaviors, which occurred only 17 months after legalization. The cannabis use frequency outcome was a crude measure that did not capture important aspects of cannabis use, such as potency or mode of use. Considering the cohort was recruited from a community-level research registry, the results may not be fully generalizable nationally or internationally, especially given the diversity of policies and regulations accompanying cannabis legalization in other jurisdictions. Moreover, high-income individuals were overrepresented in the study sample. It is also possible that residual or unmeasured confounding may have biased the results.

## Conclusions

This cohort study found a small increase in mean cannabis use frequency and a small decrease in mean cannabis misuse in the 5 years following recreational cannabis legalization in a community-based nonclinical cohort of adults in Canada. These changes were moderated by prelegalization cannabis use, with individuals with more frequent prelegalization consumption exhibiting the largest decreases in both outcomes. The apparent discrepancy between increasing cannabis use and decreasing cannabis misuse may have been driven by younger cannabis users, who typically transition from problematic to nonproblematic use as they age. This study also found that cannabis users’ product preferences evolved over the course of legalization away from dried flower and toward noncombustion products. Although longer-term follow-up is required, these results suggest a negative consequence (a small increase in cannabis use frequency) and potentially positive consequences (small decrease in cannabis misuse and transition from combustible to noncombustible cannabis products) among adults following recreational cannabis legalization in Canada.

## Notes

Supplement 1. **eTable 1.** Attrition Analysis Comparing Those Excluded Due to Having Under 2 Follow-Ups to Those Included in the Final Study Sample **eTable 2.** Sensitivity Analysis Repeating Main Analysis Linear Mixed Effects Models With Multiple Imputation Via JointAI **eTable 3.** Supplementary Linear Mixed Effects Models Examining Age and Sex as Moderators for Change in Cannabis Use Frequency and CUDIT-R in the 5 Years Following Cannabis Legalization **eFigure 1.** Flowchart of Study Cohort Exclusions and Loss to Follow-Up by Wave **eFigure 2.** Interaction Plots for All Significant Interactions for Cannabis Use Frequency and CUDIT-R Score Over 5 Years Since Legalization From September 2018 to October 2023 **eFigure 3.** Alluvial Plots Showing Transitions in Cannabis Use Frequency From Prelegalization to 5 Years Postlegalization and Across All Waves **eFigure 4.** Alluvial Plots Showing Transitions in CUDIT-R Score From Prelegalization to 5 Years Postlegalization and Across All Waves **eFigure 5.** Cannabis Product Preferences Over Time Since Cannabis Legalization Among Active Cannabis Users at Each Wave **eFigure 6.** Mean for Cannabis Use Frequency and CUDIT-R Score Over 5 Years Since Legalization From September 2018 to October 2023 Stratified by Baseline Age and Sex

Supplement 2.
Data Sharing Statement
