# Supplementary material for: Cannabis Use and Misuse Following Recreational Cannabis Legalization
Source: JAMA Netw Open. 2025 Apr 23;8(4):e256551. doi: 10.1001/jamanetworkopen.2025.6551 (PMC12019528; doi:10.1001/jamanetworkopen.2025.6551)
Supplement: Supplement 1. — eTable 1. Attrition Analysis Comparing Those Excluded Due to Having Under 2 Follow-Ups to Those Included in the Final Study Sample eTable 2. Sensitivity Analysis Repeating Main Analysis Linear Mixed Effects Models With Multiple Imputation Via JointAI eTable 3. Supplementary Linear Mixed Effects Models Examining Age and Sex as Moderators for Change in Cannabis Use Frequency and CUDIT-R in the 5 Years Following Cannabis Legalization eFigure 1. Flowchart of Study Cohort Exclusions and Loss to Follow-Up by Wave eFigure 2. Interaction Plots for All Significant Interactions for Cannabis Use Frequency and CUDIT-R Score Over 5 Years Since Legalization From September 2018 to October 2023 eFigure 3. Alluvial Plots Showing Transitions in Cannabis Use Frequency From Prelegalization to 5 Years Postlegalization and Across All Waves eFigure 4. Alluvial Plots Showing Transitions in CUDIT-R Score From Prelegalization to 5 Years Postlegalization and Across All Waves eFigure 5. Cannabis Product Preferences Over Time Since Cannabis Legalization Among Active Cannabis Users at Each Wave eFigure 6. Mean for Cannabis Use Frequency and CUDIT-R Score Over 5 Years Since Legalization From September 2018 to October 2023 Stratified by Baseline Age and Sex [file jamanetwopen-e256551-s001.pdf]

Supplemental Online Content

McDonald AJ, Doggett A, Belisario K, et al. Cannabis use and misuse following recreational cannabis legalization. *JAMA Netw Open*. 2025;8(4):e256551. doi:10.1001/jamanetworkopen.2025.6551

eTable 1. Attrition Analysis Comparing Those Excluded Due to Having Under 2 Follow-Ups to Those Included in the Final Study Sample
eTable 2. Sensitivity Analysis Repeating Main Analysis Linear Mixed Effects Models With Multiple Imputation Via JointAI
eTable 3. Supplementary Linear Mixed Effects Models Examining Age and Sex as Moderators for Change in Cannabis Use Frequency and CUDIT-R in the 5 Years Following Cannabis Legalization
eFigure 1. Flowchart of Study Cohort Exclusions and Loss to Follow-Up by Wave
eFigure 2. Interaction Plots for All Significant Interactions for Cannabis Use Frequency and CUDIT-R Score Over 5 Years Since Legalization From September 2018 to October 2023
eFigure 3. Alluvial Plots Showing Transitions in Cannabis Use Frequency From Prelegalization to 5 Years Postlegalization and Across All Waves
eFigure 4. Alluvial Plots Showing Transitions in CUDIT-R Score From Prelegalization to 5 Years Postlegalization and Across All Waves
eFigure 5. Cannabis Product Preferences Over Time Since Cannabis Legalization Among Active Cannabis Users at Each Wave
eFigure 6. Mean for Cannabis Use Frequency and CUDIT-R Score Over 5 Years Since Legalization From September 2018 to October 2023 Stratified by Baseline Age and Sex

This supplemental material has been provided by the authors to give readers additional information about their work.

© 2025 McDonald AJ et al. *JAMA Network Open*.

eTable 1: Attrition analysis comparing those excluded due to having under 2 follow-ups to those included in the final study sample.

	Included (n=1,428)	Lost to Follow-up (n=51)	X^2/t-test p-value
**Baseline characteristic**			
Sex			0.148
Female	859 (60.2%)	25 (49.0%)	
Male	569 (39.8%)	26 (51.0%)	
Age			
Mean (SD)	34.5 (13.9)	36.1 (14.4)	0.426
Ethnicity			0.151
White	1127 (78.9%)	45 (88.2%)	
Additional racial and ethnic groups	301 (21.1%)	6 (11.8%)	
Marital status			0.929
Unmarried	975 (68.3%)	34 (66.7%)	
Married	453 (31.7%)	17 (33.3%)	
Household income			0.011
Less than $45,000	442 (31.0%)	17 (33.3%)	
$45,000 to $90,000	437 (30.6%)	24 (47.1%)	
$90,000+	549 (38.4%)	10 (19.6%)	
Education			0.109
Post-secondary degree	653 (45.7%)	17 (33.3%)	
No post-secondary degree	775 (54.3%)	34 (66.7%)	
Cannabis use frequency			0.024
Never	749 (51.6%)	21 (41.2%)	
Less than monthly	246 (17.0%)	6 (11.8%)	
Monthly	168 (11.6%)	5 (9.8%)	
Weekly	141 (9.7%)	10 (19.6%)	
Daily or more	124 (8.5%)	9 (17.6%)	
Cannabis misuse (CUDIT-R score)			0.218
Less than 6	1199 (84.0%)	39 (76.5%)	
6 or more	229 (16.0%)	12 (23.5%)	

Notes: CUDIT-R = Cannabis Use Disorder Identification Test – Revised.

© 2025 McDonald AJ et al. *JAMA Network Open*.

eTable 2: Sensitivity analysis repeating main analysis linear mixed effects models with multiple imputation via JointAI.

	Outcome 1: Cannabis use frequency (Percentage of days using cannabis)	Outcome 2: Cannabis misuse (CUDIT-R score)
	b (95% CI)	*b (95% CI)*
	Main effect models	
Time (in years)	0.34 (0.18 to 0.50)	-0.08 (-0.10 to -0.06)
	Interaction models	
Time (in years)	0.80 (0.58 to 1.02)	0.10 (0.08 to 0.12)
Pre-legalization cannabis use		
None	Ref	Ref
Less than monthly	1.83 (-0.70 to 4.36)	1.49 (1.14 to 1.84)
Monthly	9.06 (6.12 to 12.00)	3.11 (2.68 to 3.54)
Weekly	36.79 (33.69 to 39.89)	6.08 (5.63 to 6.53)
Daily	91.22 (87.89 to 94.55)	10.12 (9.65 to 10.59)
Time* Pre-legalization cannabis use		
Time*Less than monthly	0.43 (0.00 to 0.86)	-0.21 (-0.27 to -0.15)
Time*Monthly	0.61 (0.12 to 1.10)	-0.30 (-0.36 to -0.24)
Time*Weekly	-1.01 (-1.56 to -0.46)	-0.48 (-0.56 to -0.40)
Time* Daily	-6.14 (-6.17 to -5.57)	-0.78 (-0.86 to -0.70)

Notes: CUDIT-R = Cannabis Use Disorder Identification Test – Revised. All models adjusted for baseline age, sex, ethnicity, income, education, and marital status.

© 2025 McDonald AJ et al. *JAMA Network Open*.

eTable 3: Supplementary linear mixed effects models examining age and sex as moderators for change in cannabis use frequency and CUDIT-R in the 5 years following cannabis legalization.

	Outcome 1: Cannabis use frequency (Percentage of days using cannabis)			Outcome 2: Cannabis misuse (CUDIT-R score)		
		Omnibus test			Omnibus test	
	b (95% CI)	*F*	p	*b (95% CI)*	*F*	p
Time (in years)	0.29 (0.02 to 0.56)	**18.56**	**<0.001**	-0.12 (-0.14 to -0.10)	**54.54**	**<0.001**
Baseline age (30+)	-0.20 (-2.38 to 1.98)	0.03	0.858	-0.55 (-0.86 to -0.24)	**12.37**	**<0.001**
Time*Baseline age	0.11 (-0.20 to 0.42)	0.49	0.486	0.09 (0.05 to 0.13)	**19.39**	**<0.001**
Time (in years)	0.44 (0.24 to 0.64)	**15.03**	**<0.001**	-0.05 (-0.07 to -0.03)	**59.98**	**<0.001**
Sex (Male)	0.97 (-0.91 to 2.85)	1.03	0.311	0.51 (0.26 to 0.76)	**14.75**	**<0.001**
Time*Sex	-0.25 (-0.56 to 0.06)	2.37	0.124	-0.06 (-0.10 to -0.02)	**7.74**	**<0.001**
Time (in years)	0.56 (-0.48 to 1.60)	2.10	0.148	-0.08 (-0.21 to 0.06)	**5.64**	**0.018**
COVID-19 pandemic		1.12	0.328		**5.92**	**0.003**
Pre-pandemic (Sept 2018 to Oct 2019)	Ref			Ref		
Peak pandemic (Apr 2020 to Oct 2021)	1.41 (-0.48 to 3.31)			0.20 (-0.05 to 0.44)		
Late/post-pandemic (Apr 2022 to Oct 2023)	0.71 (-2.64 to 4.05)			-0.64 (-1.07 to -0.21)		
Time*COVID-19 pandemic		0.13	0.879		**9.54**	**<0.001**
Peak pandemic (Apr 2020 to Oct 2021)	-0.31 (-1.59 to 0.97)			-0.15 (-0.31 to 0.01)		
Late and post-pandemic (Apr 2022 to Oct 2023)	-0.30 (-1.57 to 0.97)			0.15 (-0.02 to 0.31)		

Notes: CUDIT-R = Cannabis Use Disorder Identification Test – Revised. All models adjusted for baseline age, sex, ethnicity, income, education, and marital status. When testing age as a moderator, we dichotomized age to separate young adults, who typically age out of cannabis use,[30] from middle-aged and older adults. eFigure 6 illustrates all significant interactions facilitating interpretation.

© 2025 McDonald AJ et al. *JAMA Network Open.*

eFigure 1: Flowchart of study cohort exclusions and loss to follow up by wave.

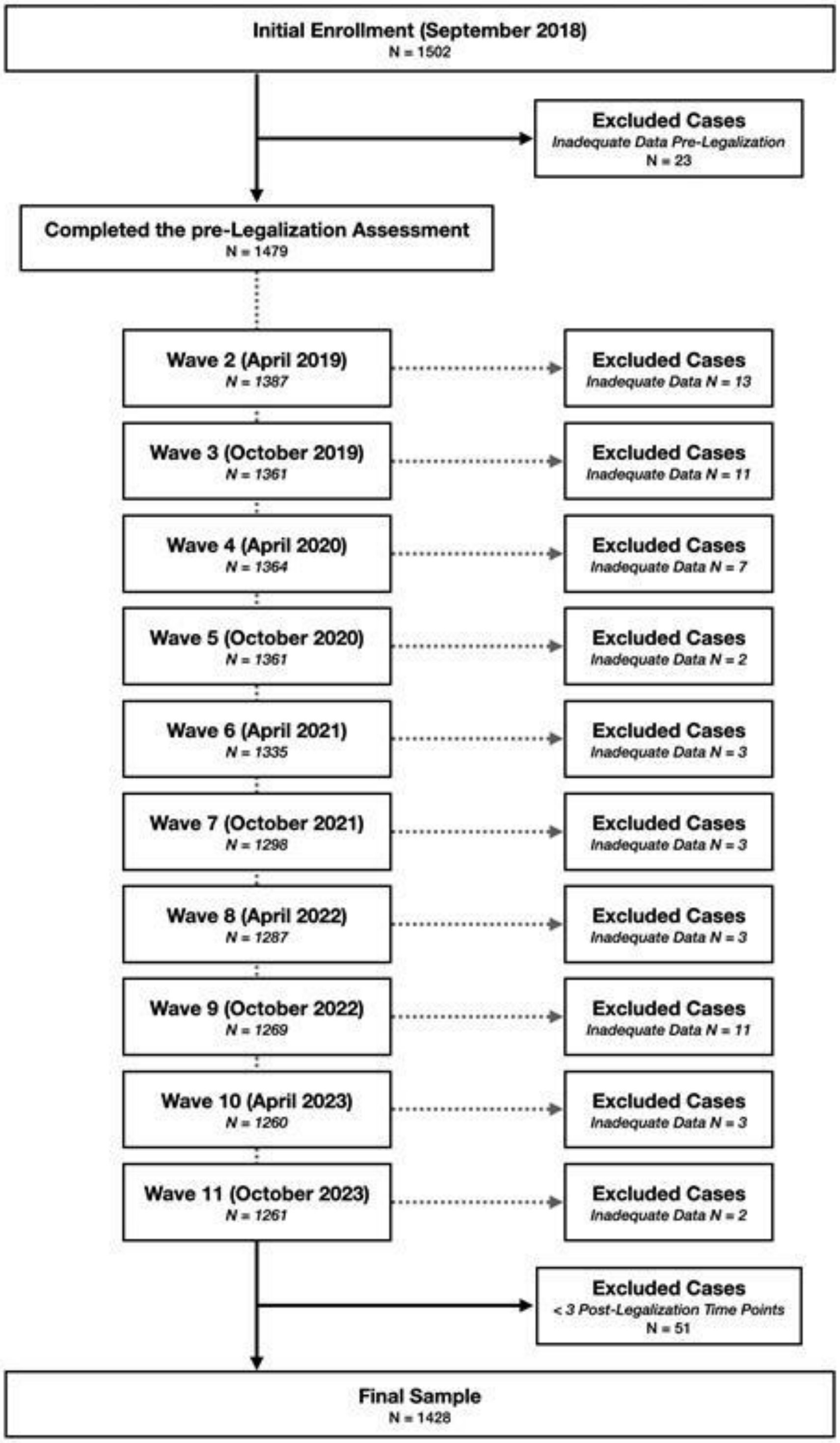

© 2025 McDonald AJ et al. *JAMA Network Open*.

eFigure 2: Interaction plots for all significant interactions for cannabis use frequency and CUDIT-R score over 5 years since legalization from September 2018 to October 2023 (10 waves).

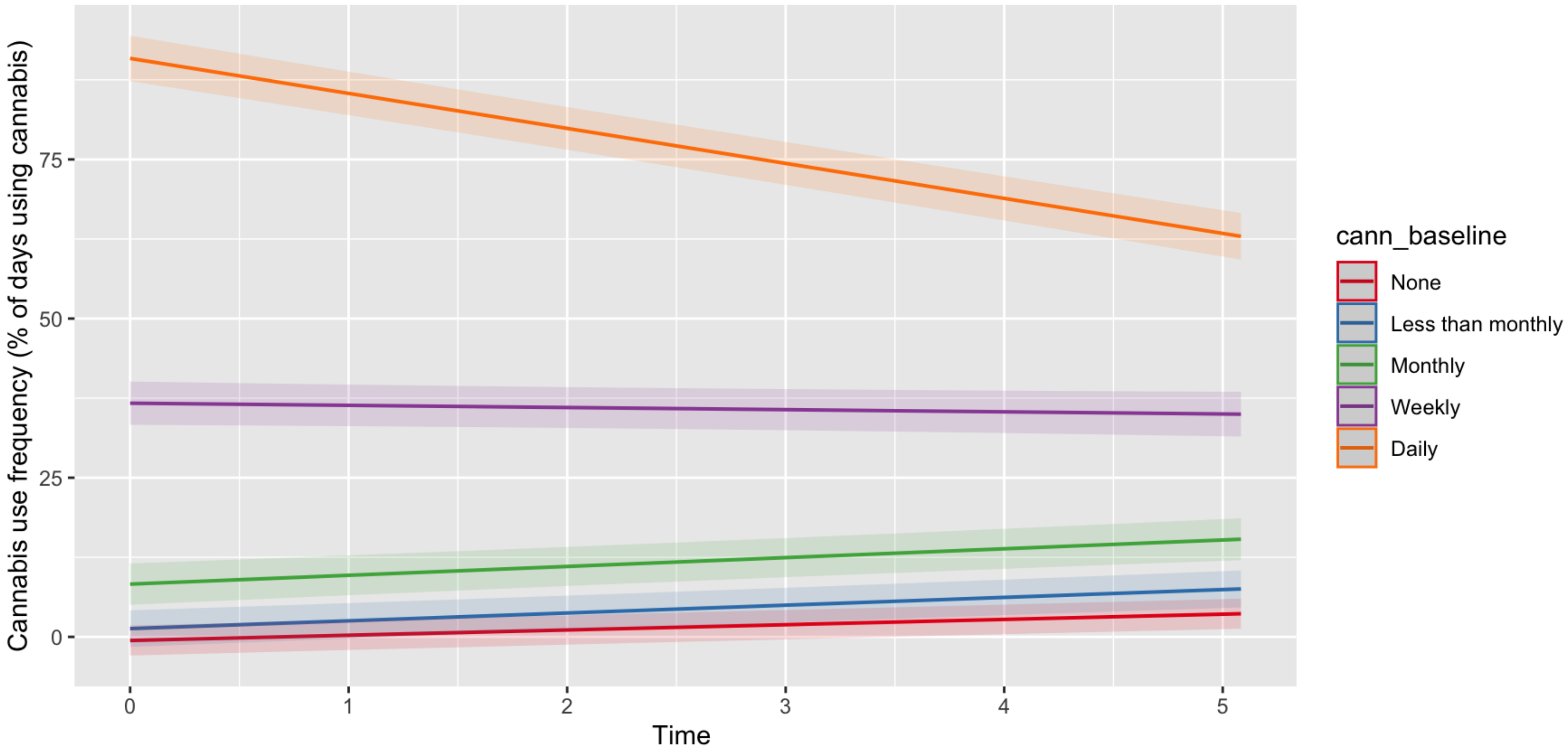

A: Interaction between baseline cannabis use frequency and time with cannabis use frequency (% of days using cannabis) as the outcome

© 2025 McDonald AJ et al. *JAMA Network Open*.

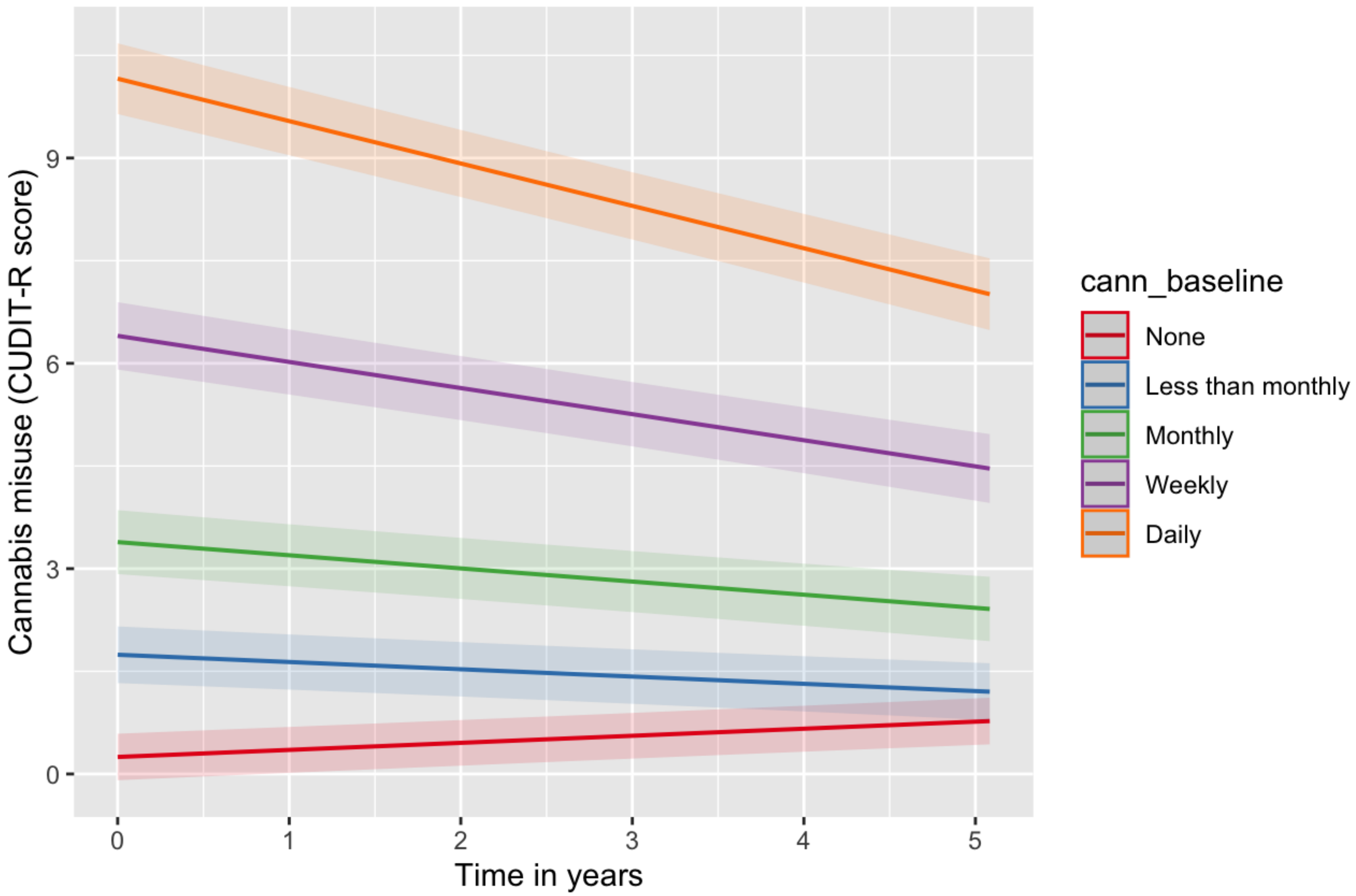

B: Interaction between baseline cannabis use frequency and time with cannabis misuse (CUDIT-R score) as the outcome

© 2025 McDonald AJ et al. *JAMA Network Open*.

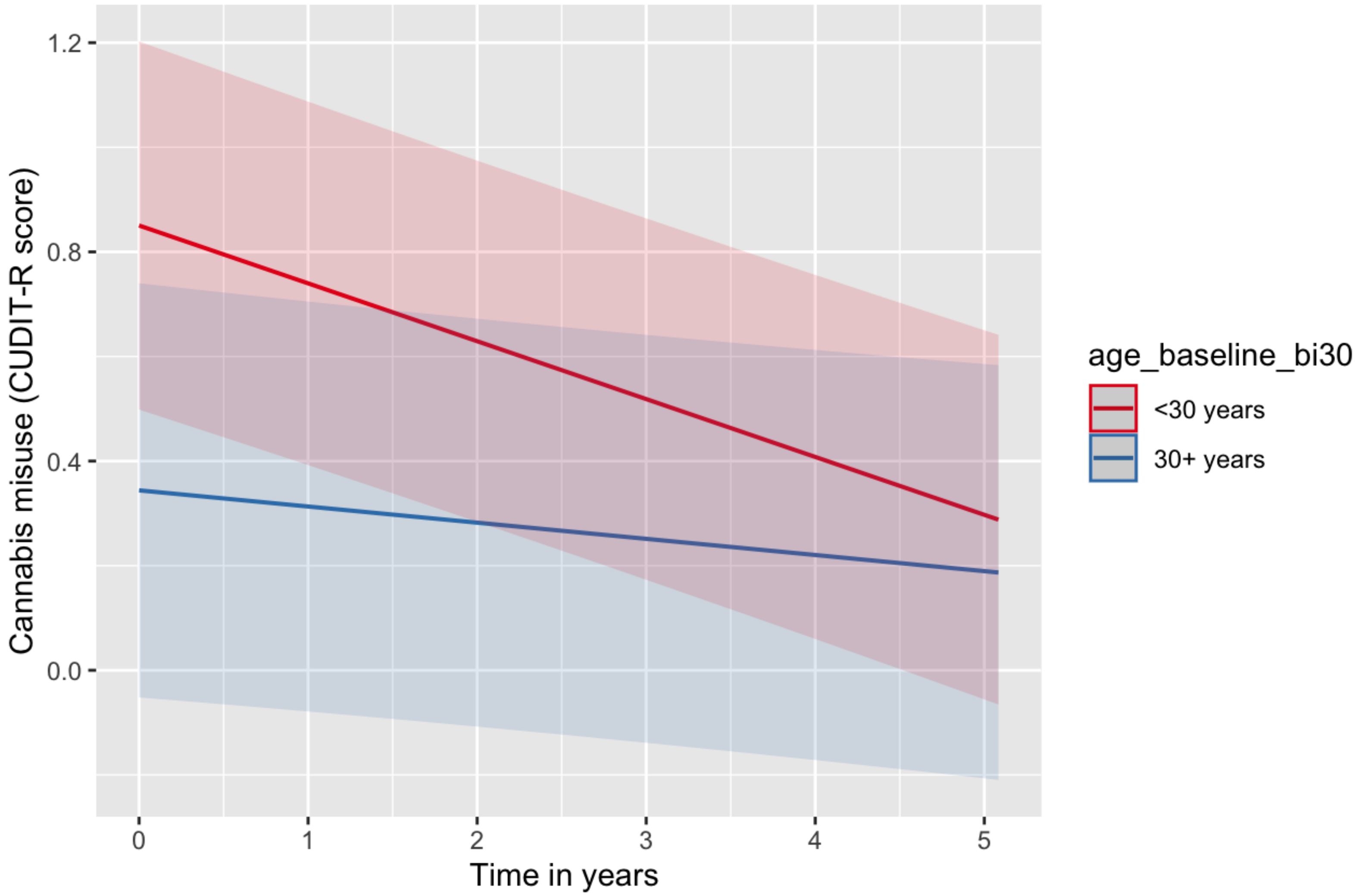

C: Interaction between baseline age and time with cannabis misuse (CUDIT-R score) as the outcome

© 2025 McDonald AJ et al. *JAMA Network Open*.

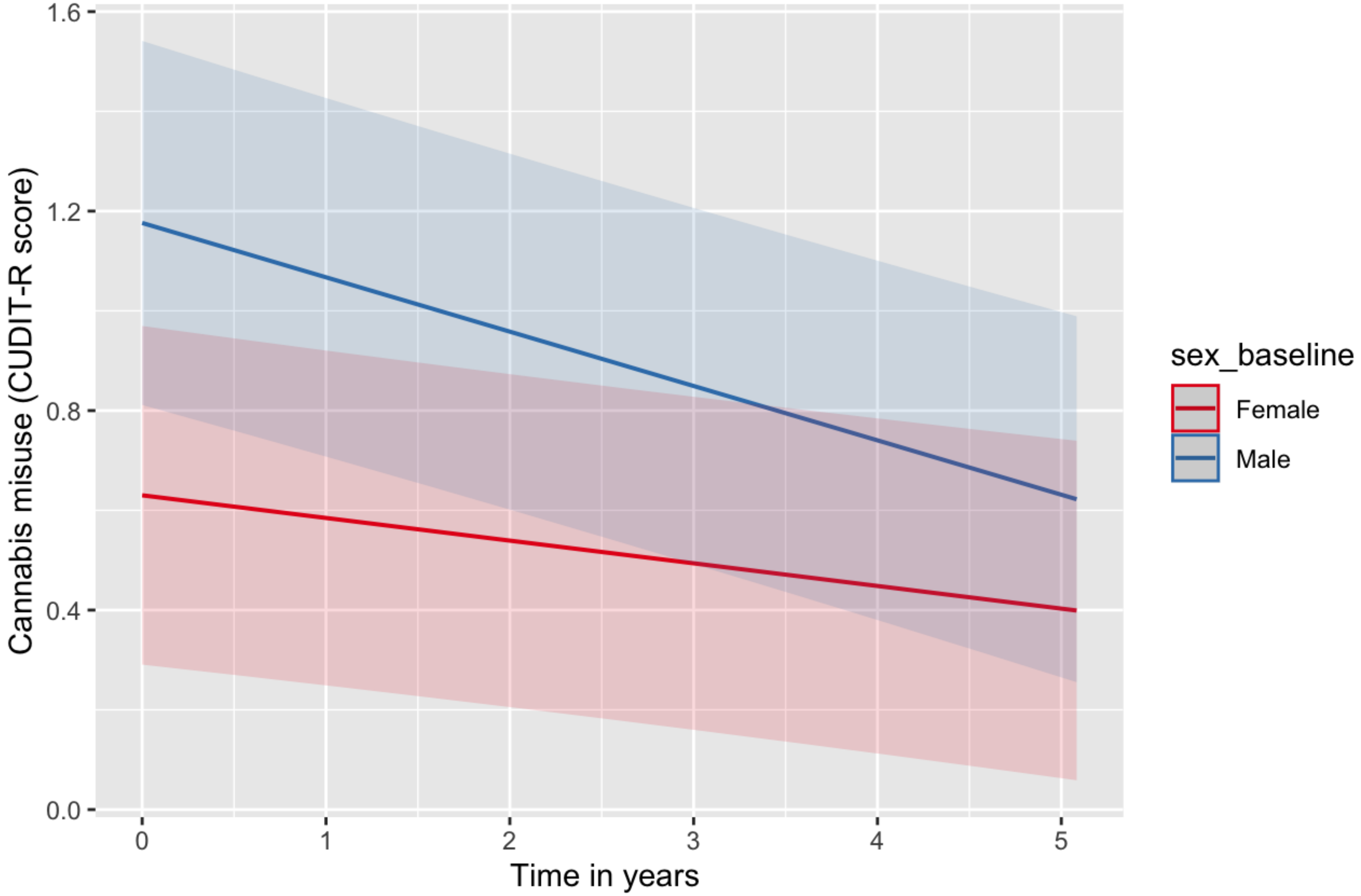

D: Interaction between sex and time with cannabis misuse (CUDIT-R score) as the outcome

© 2025 McDonald AJ et al. *JAMA Network Open*.

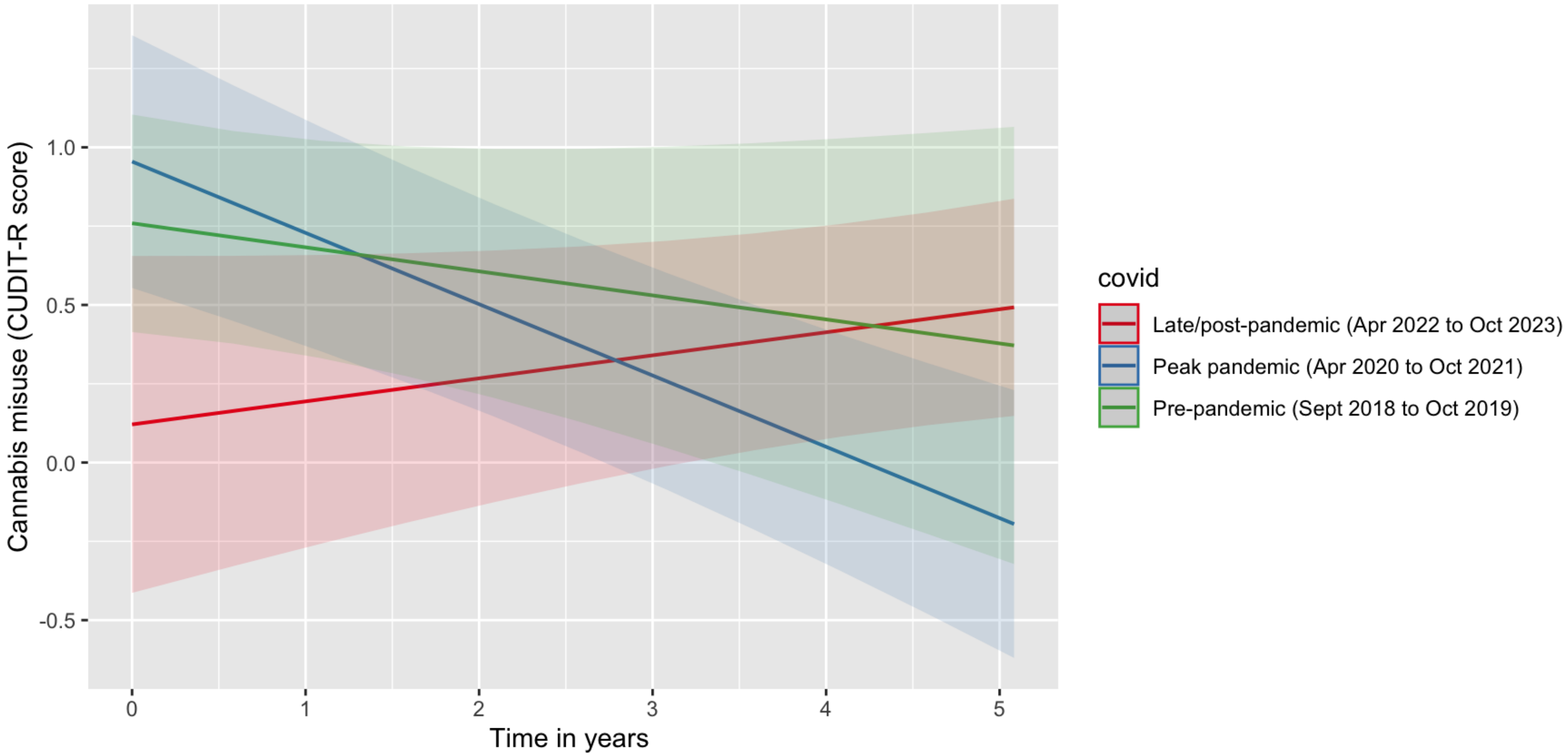

E: Interaction between COVID-19 pandemic period and time with cannabis misuse (CUDIT-R score) as the outcome

Notes: All interaction plots were estimated based on linear mixed models, assuming all adjusted covariates held at reference values (females, 19 years of age, no cannabis use at baseline, white, <$45,000 income, bachelor's degree, and unmarried).

© 2025 McDonald AJ et al. *JAMA Network Open*.

eFigure 3: Alluvial plots showing transitions in cannabis use frequency from A) pre-legalization (September 2018) to 5 years post-legalization (October 2023) and B) across all waves.

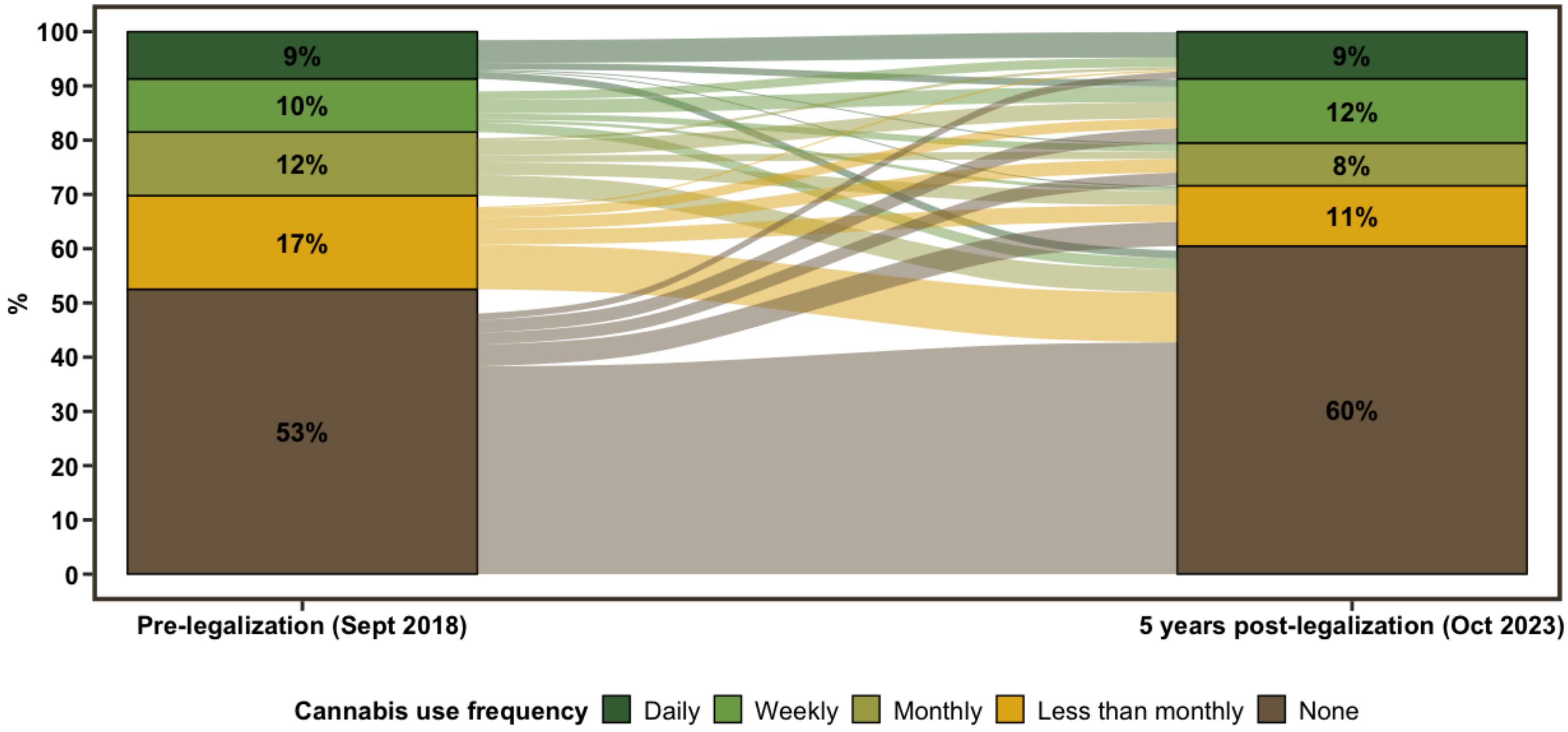

A: Pre-legalization to 5 years post-legalization

© 2025 McDonald AJ et al. *JAMA Network Open*.

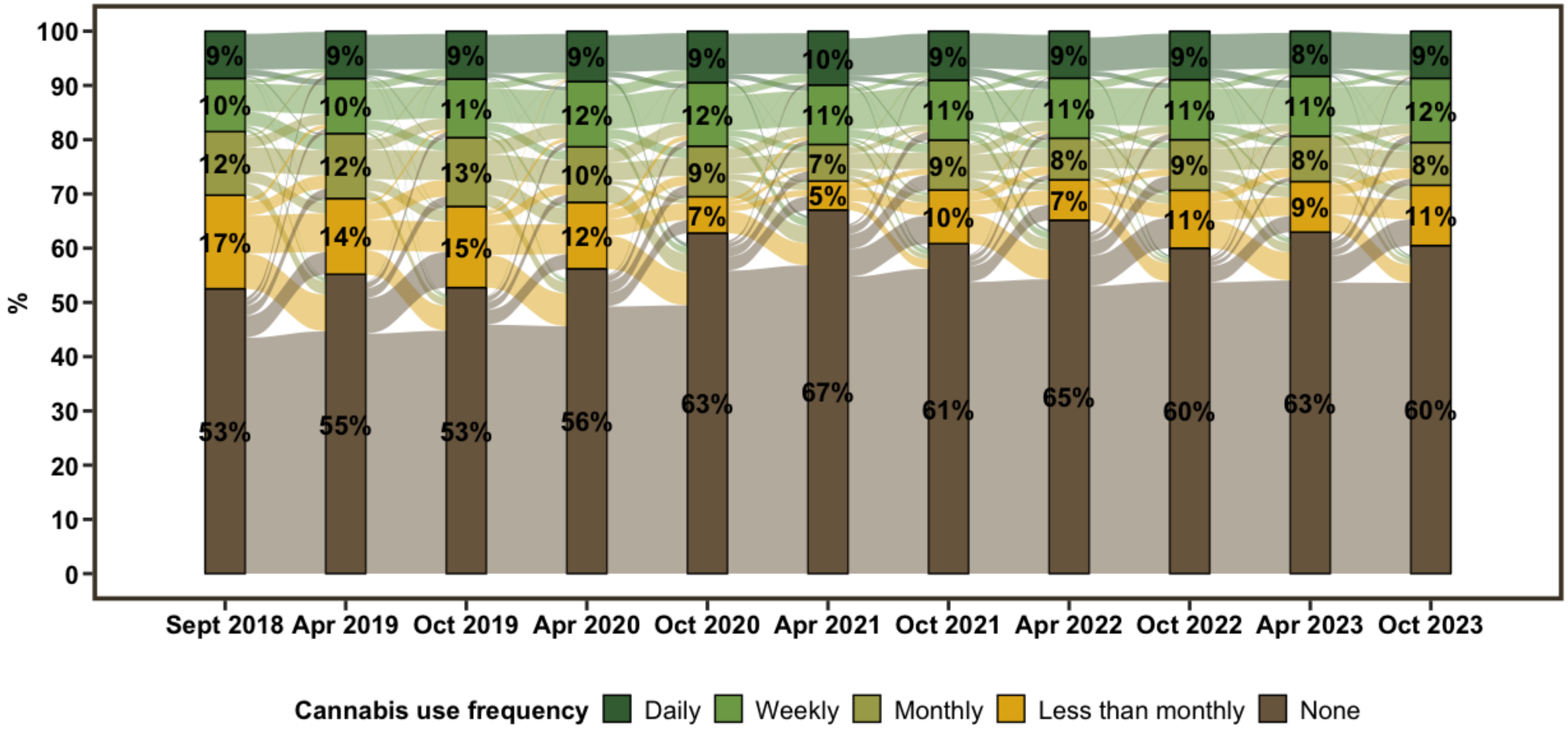

B: All waves across 5 years

© 2025 McDonald AJ et al. *JAMA Network Open*.

eFigure 4: Alluvial plots showing transitions in CUDIT-R score from A) pre-legalization (September 2018) to 5 years post-legalization (October 2023) and B) across all waves.

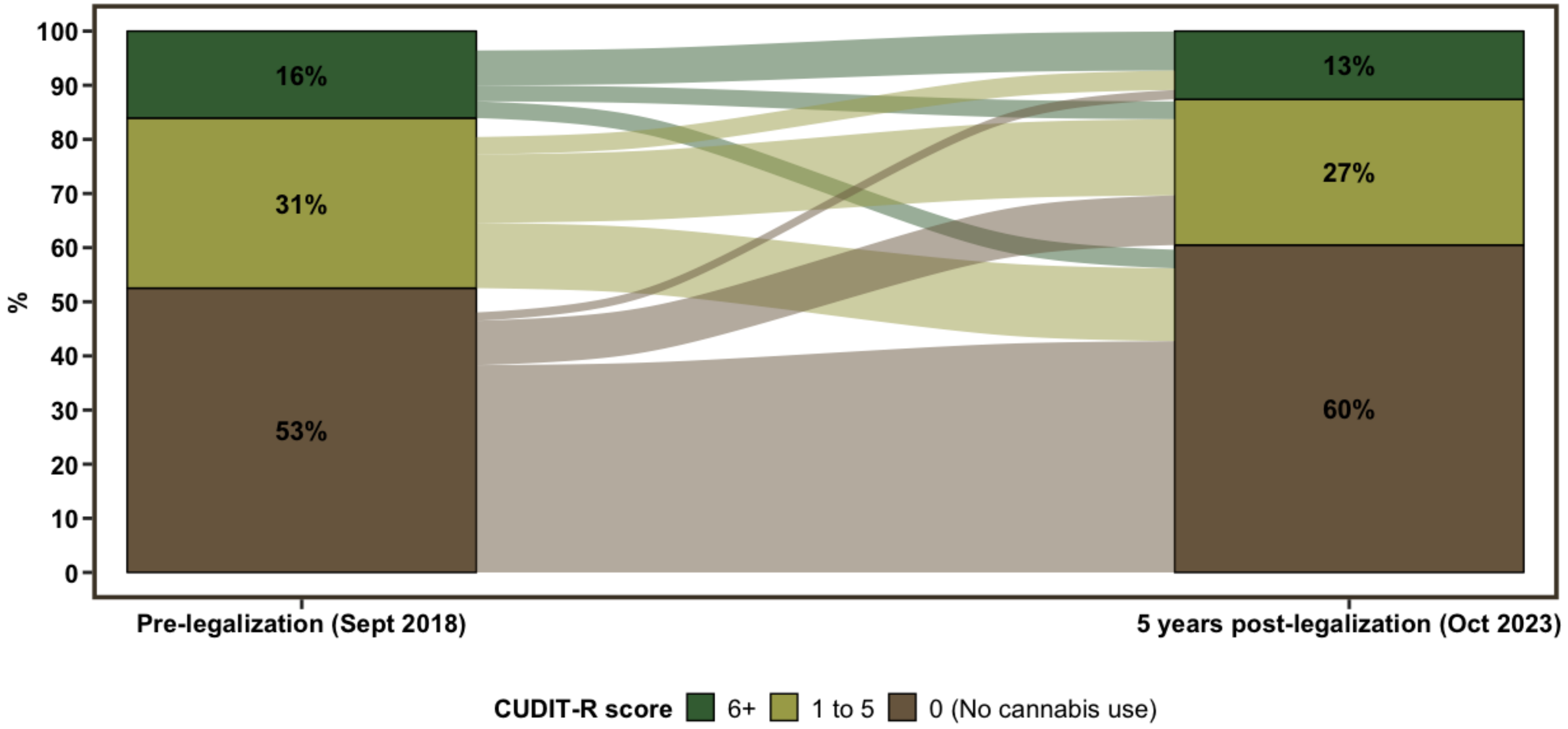

A: Pre-legalization to 5 years post-legalization

© 2025 McDonald AJ et al. *JAMA Network Open*.

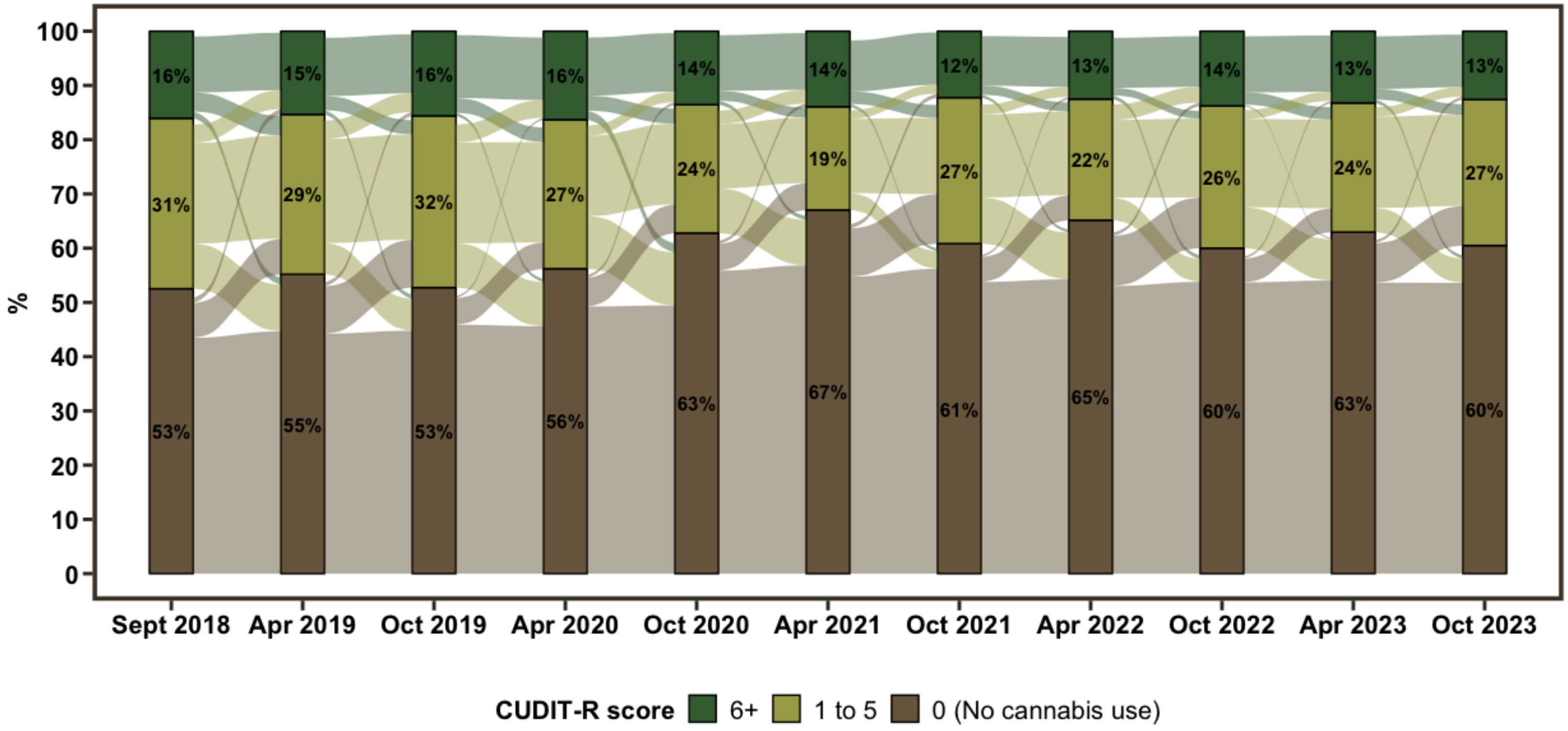

B: All waves across 5 years

Note: CUDIT-R = Cannabis Use Disorder Identification Test – Revised

© 2025 McDonald AJ et al. *JAMA Network Open*.

eFigure 5: Cannabis product preferences over time since cannabis legalization among active cannabis users at each wave

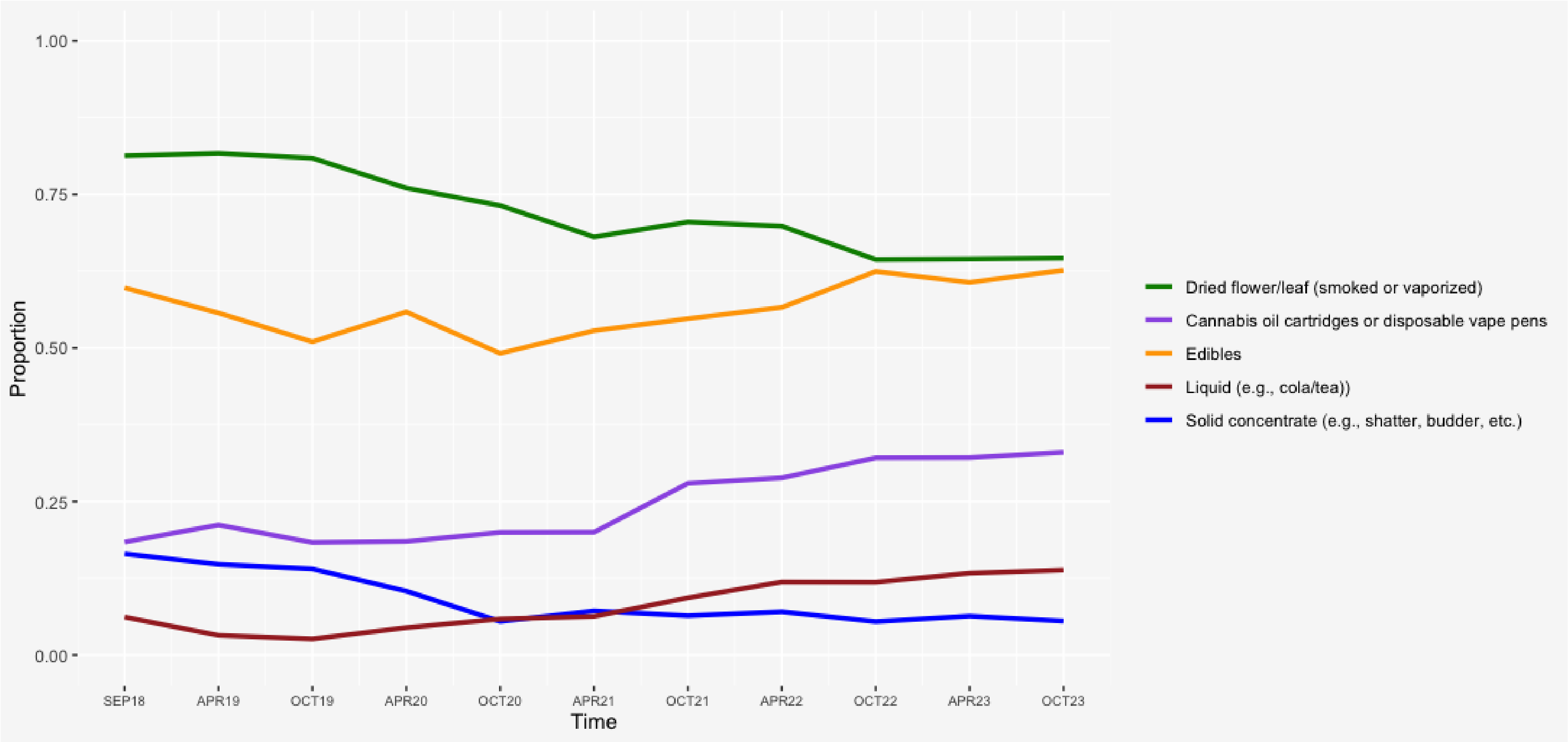

A: Cannabis product preferences that changed most since legalization

© 2025 McDonald AJ et al. *JAMA Network Open*.

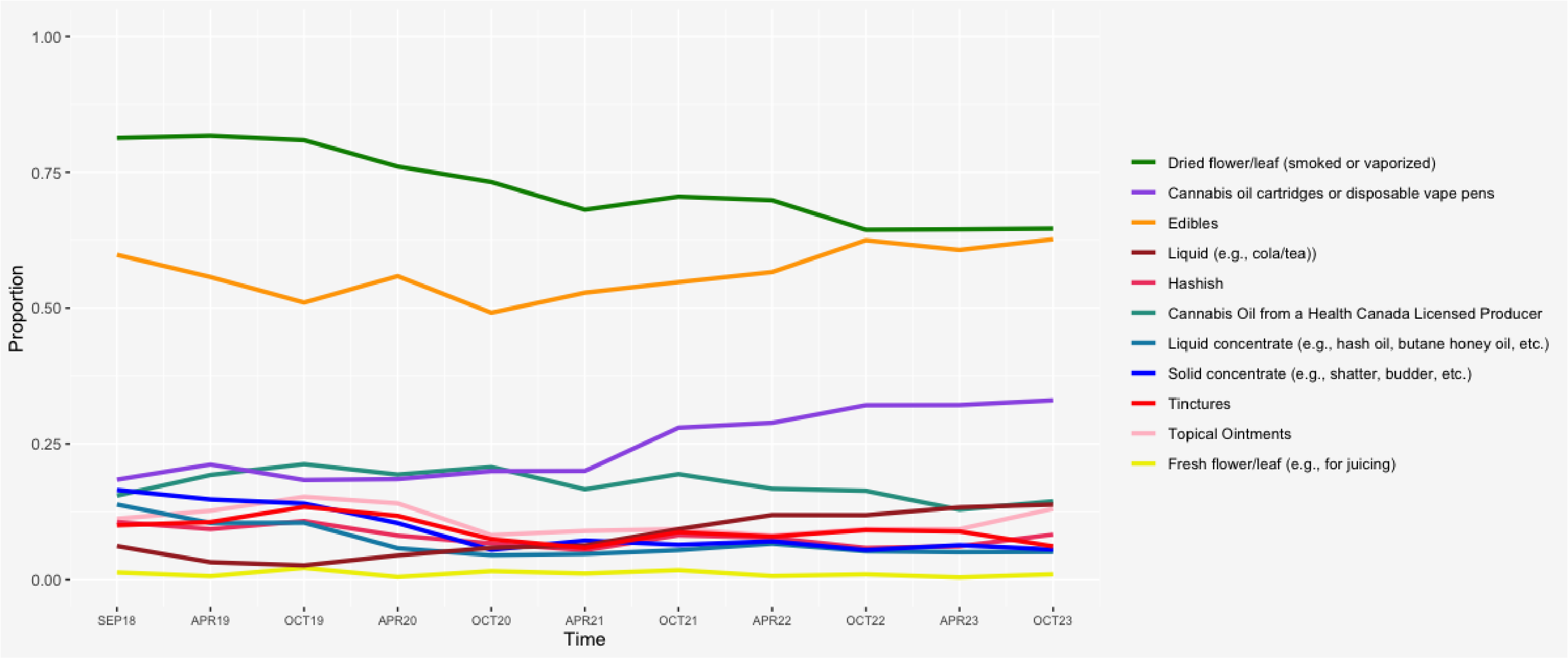

B: All products

© 2025 McDonald AJ et al. *JAMA Network Open*.

eFigure 6: Mean (±SEM) for cannabis use frequency and CUDIT-R score over 5 years since legalization from September 2018 to October 2023 (10 waves) stratified by baseline age (subpanels A and C) and sex (subpanels B and D).

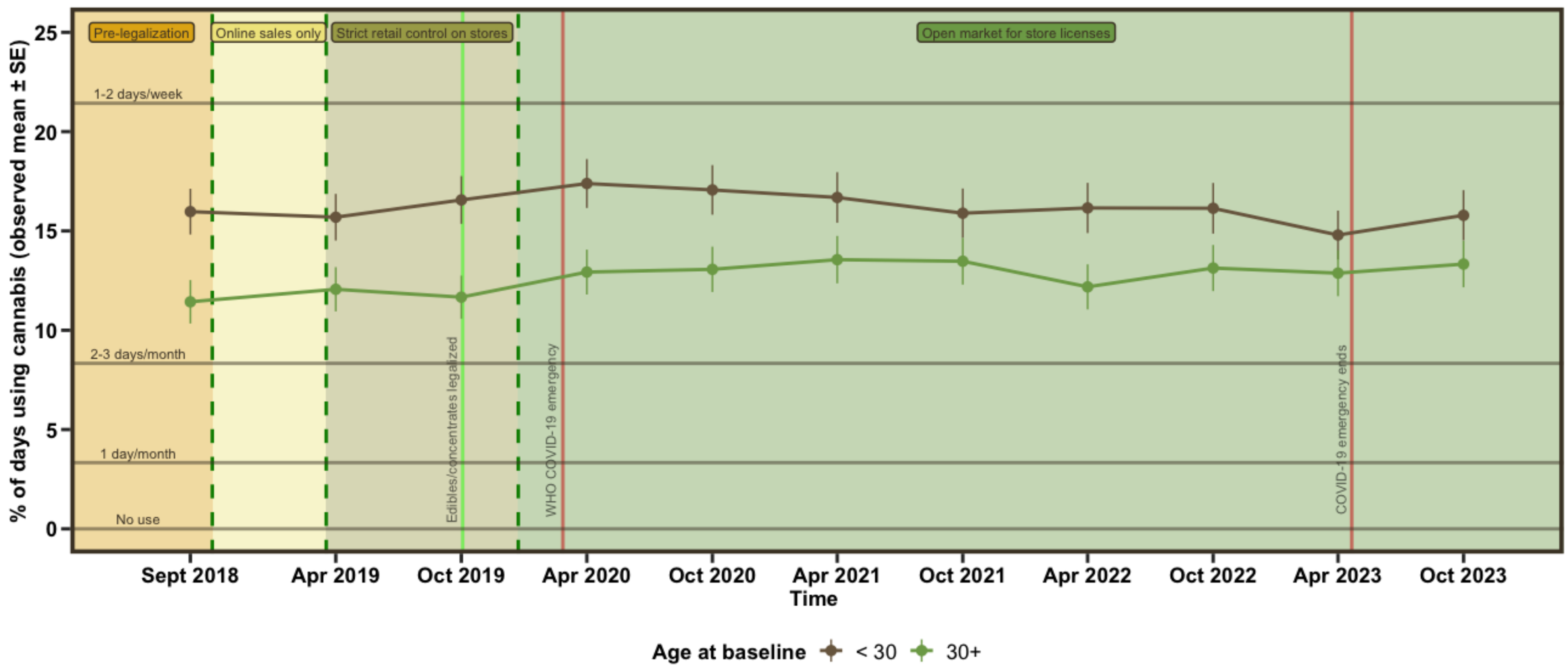

A: Changes in cannabis use frequency stratified by age

© 2025 McDonald AJ et al. *JAMA Network Open*.

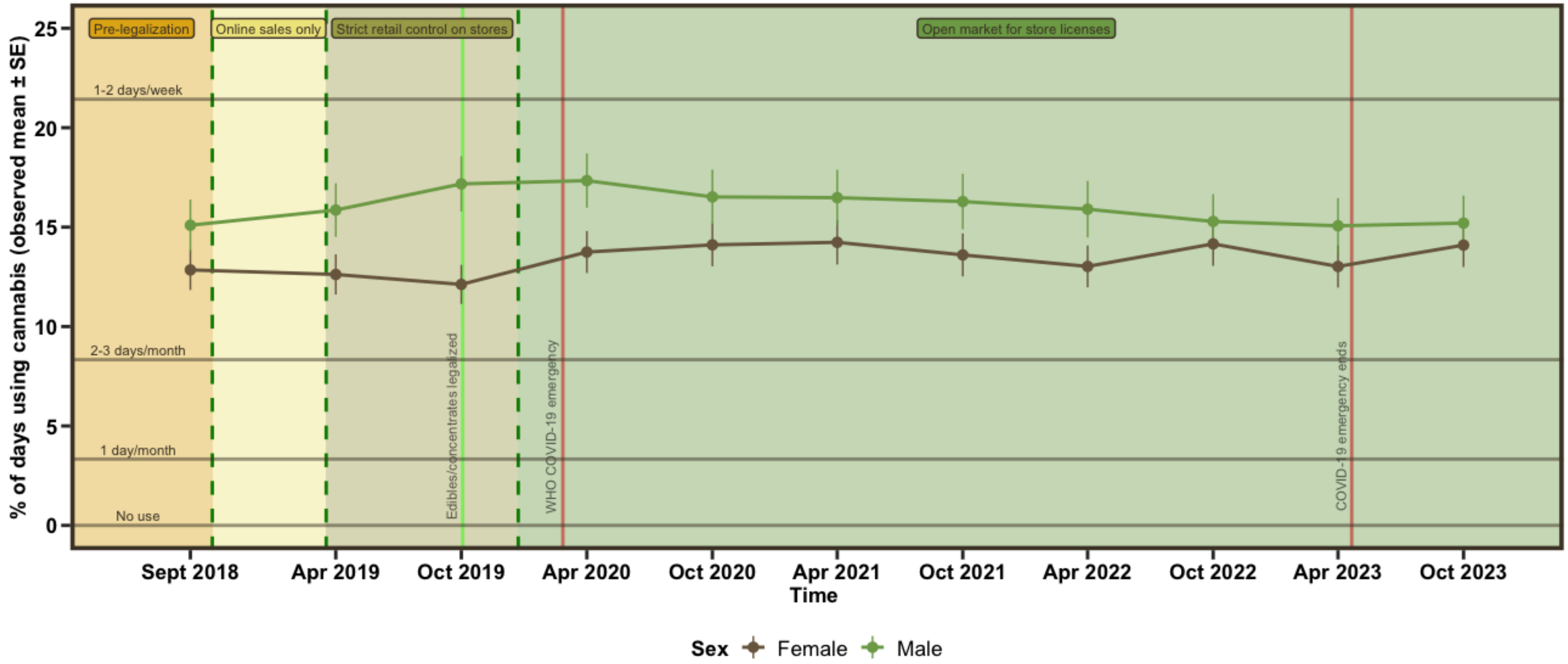

B: Changes in cannabis use frequency stratified by sex

© 2025 McDonald AJ et al. *JAMA Network Open*.

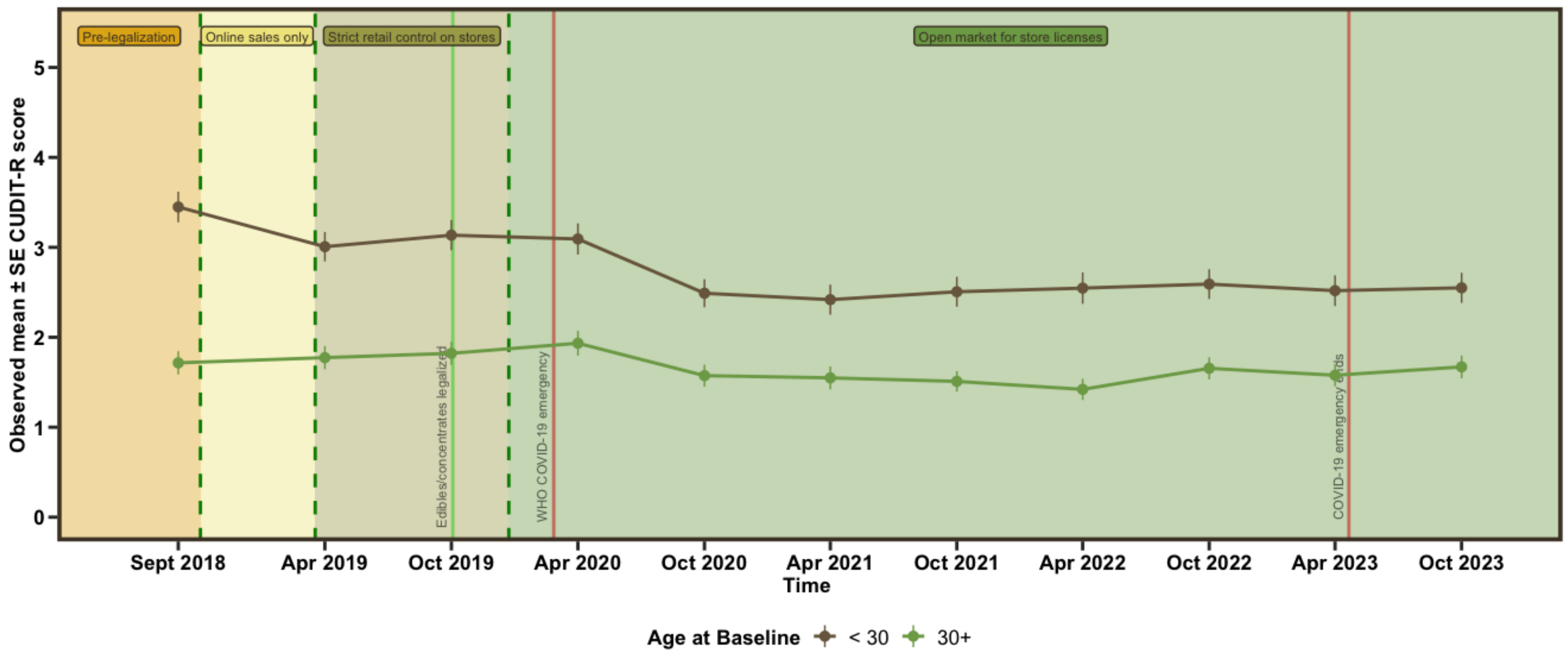

C: Changes in CUDIT-R score stratified by age

© 2025 McDonald AJ et al. *JAMA Network Open*.

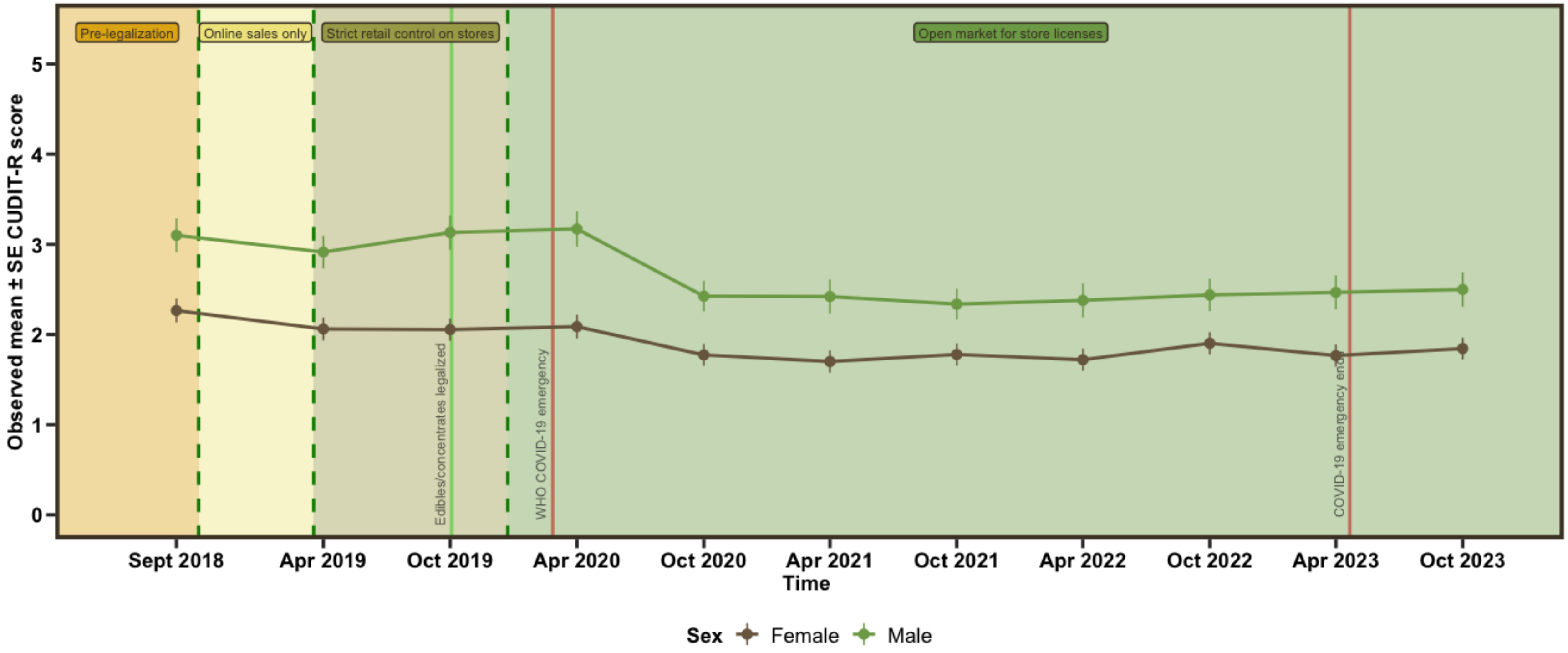

D: Changes in CUDIT-R score stratified by sex

Notes: CUDIT-R = Cannabis Use Disorder Identification Test – Revised

© 2025 McDonald AJ et al. *JAMA Network Open*.
