# Supplementary material for: Cannabis Use and Misuse Following Recreational Cannabis Legalization
Source: JAMA Netw Open. 2025 Apr 23;8(4):e256551. doi: 10.1001/jamanetworkopen.2025.6551 (PMC12019528; doi:10.1001/jamanetworkopen.2025.6551)
Supplement: Supplement 2. — Data Sharing Statement [file jamanetwopen-e256551-s002.pdf]

Data Sharing Statement

McDonald. Cannabis Use and Misuse Following Recreational Cannabis Legalization. *JAMA Netw Open*. Published April 23, 2025. doi:10.1001/jamanetworkopen.2025.6551

Data
Data available: Yes
Data types: Deidentified participant data, Data dictionary
How to access data: Data from this study will be made available upon request to qualified researchers with appropriate goals for further analysis and subject to PI and research ethics board approval.
When available: With publication

Supporting Documents
Document types: Statistical/analytic code, Informed consent form
How to access documents: Supporting documents are available upon request to qualified researchers with appropriate goals for further analysis and subject to PI and research ethics board approval.
When available: With publication

Additional Information
Who can access the data: Researchers whose proposed use of the data has been approved.
Types of analyses: For an approved purpose.
Mechanisms of data availability: After approval of a proposal.
